# Development, feasibility, and acceptability of a smartphone-based ecological momentary assessment of minority stress and suicidal ideation among sexual and gender minority youth

**DOI:** 10.1371/journal.pone.0330204

**Published:** 2025-08-12

**Authors:** Kirsty Clark, Kaitlyn Phillips, Elisa Park, Alexandra Argiros, Alexandros Nikolaidis-Konstas, Joseph Sexton, Melissa Cyperski, Evan Kleiman, John Pachankis

**Affiliations:** 1 Department of Medicine, Health, and Society, Vanderbilt University, Nashville, Tennessee, United States of America; 2 Department of Psychology and Human Development, Vanderbilt University, Nashville, Tennessee, United States of America; 3 Department of Psychology, McGill University, Montreal, Quebec, Canada; 4 Department of Psychiatry and Behavioral Sciences, Vanderbilt University Medical Center, Nashville, Tennessee, United States of America; 5 Department of Psychology, Rutgers, The State University of New Jersey, New Brunswick, New Jersey, United States of America; 6 Department of Social and Behavioral Sciences, Yale School of Public Health, New Haven, Connecticut, United States of America; Universiteit Antwerpen, BELGIUM

## Abstract

**Objective:**

We sought to develop and assess the feasibility and acceptability of a smartphone-based ecological momentary assessment (EMA) study of minority stress and suicidal ideation intensity among sexual and gender minority youth (SGMY) in the US Southeast.

**Methods:**

In Study 1, the EMA protocol was developed through an iterative process, incorporating qualitative input from focus groups and interviews with 16 parents of SGMY and 16 SGMY from the US Southeast as well as six clinicians and researchers. In Study 2, 50 SGMY aged 13–24 with past-year suicidal ideation and current depressive symptoms were recruited from the US Southeast. The study included a baseline assessment, 28 consecutive days of EMA surveys (3x per day), a weekly acceptability survey, and a post-study exit interview.

**Results:**

In Study 1, qualitative feedback guided the selection, adaptation, and development of EMA measures and informed study features including the EMA schedule, reminder notifications, incentive structure, and the safety and risk monitoring protocol. In Study 2, the EMA protocol demonstrated feasibility through high compliance with the EMA survey (**M* *= 80.21%, *SD *= 16.92%, **Mdn* *= 83.93%, range* = *38.10%−100.00%) with some variation over time and by participant age. Weekly feedback surveys indicated high acceptability, with participants reporting that the EMA surveys were easy to complete and private, understandable, minimally burdensome, and at least moderately engaging. Exit interviews revealed several themes, including facilitators of high engagement, barriers to engagement, intervention implications, and suggested improvements for future EMA studies.

**Conclusions:**

Smartphone-based EMA is a feasible and acceptable method for studying real-time experiences of minority stress and suicidal ideation intensity among SGMY at high risk. Incorporating community member feedback during EMA study development can help to ensure cultural responsiveness and enhance participant compliance. This paper provides practical guidance for researchers planning to conduct EMA suicide research with SGMY.

## Introduction

A substantially higher proportion of sexual and gender minority youth (SGMY) experience suicidal ideation compared to their cisgender and heterosexual peers [1,2]. Recent national survey data from the Centers for Disease Control and Prevention reveals that approximately 47% of lesbian, gay, and bisexual, as well as 44% of transgender high school students have seriously contemplated suicide within the past year, in sharp contrast to about 15% of their heterosexual, cisgender counterparts [1,2]. Research indicates that the increased likelihood of suicidal ideation among SGMY is linked to minority stress, which refers to the excess stress faced by individuals with minoritized identities [3,4]. According to minority stress theory, exposure to distal minority stressors (e.g., rejection, discrimination) intensifies negative psychological responses to stigma, known as proximal minority stressors (e.g., internalized stigma, hypervigilance). These proximal stressors, along with universal precursors to suicide (e.g., hopelessness, thwarted belongingness), contribute to the disproportionately high risk of suicidal ideation and suicide attempts among SGMY [5].

Research linking minority stress to suicidal ideation among SGMY has predominantly relied on observational, cross-sectional data which assesses suicidal ideation retrospectively at a single time point [5–7]. Such single time point measures assessing suicidal ideation (e.g., “Have you ever seriously considered suicide?”) are subject to recall bias and obscure more granular information on the momentary fluctuations of suicidal ideation and its mechanisms [8,9]. An emerging body of research shows that suicidal ideation demonstrates significant within-person heterogeneity (i.e., variability in the intensity and frequency of suicidal thoughts experienced by an individual over time), an episodic nature, and a brief duration [10,11]. Notably, in the most comprehensive study to date on the timescale of suicidal ideation, which was conducted without regard for SGM status, authors found that elevated suicidal ideation lasts on average one to three hours before subsiding [8]. Thus, the current literature on the relationship between minority stress and suicidal ideation in SGMY, which is primarily based on cross-sectional and retrospective assessments, fails to capture the minority stress-to-suicidal ideation cascade as it unfolds in real-time and in everyday life, hampering understanding of how within-person variations in exposure to minority stress may be linked to short-term risk of suicidal ideation intensity among SGMY.

Ecological momentary assessment (EMA) may be a particularly valuable methodology for investigating the dynamic influence of minority stress on the suicidal ideation intensity of SGMY within their everyday lives. EMA typically requires participants to respond to multiple surveys throughout the day over a specific period, effectively minimizing recall bias by capturing participants’ real-time experiences [12,13]. Leveraging the widespread use of smartphones provides a cost-effective and practical means for monitoring real-time experiences through EMA [10,14–16]. Yet while EMA is increasingly acknowledged as a valuable method for capturing short-term fluctuations (e.g., across hours) in suicidal ideation and its precursors, there exists a notable lack of consistency in the construction of EMA studies [17–19]. For example, EMA studies assessing suicidal ideation demonstrate substantial variation across key elements of study protocols, including sampling frequency (the number of surveys per day), study duration (the number of days surveys are deployed), and the constructs assessed (exposures, outcomes, and mechanisms) [13,17]. Even when studies focus on the same constructs, such as suicidal ideation intensity, there are substantial discrepancies in assessment methods and the number of items employed to measure a given construct. EMA studies also frequently exhibit considerable variation in many of the logistical aspects key to their implementation, including incentive structures, technical feedback mechanisms, and safety and risk monitoring protocols initiated in response to high-risk reports [13,20,21]. The latter element is a particularly pertinent ethical and logistical consideration in EMA studies that assess real-time suicidal ideation intensity to ensure participant safety during the study period.

A handful of prior studies have used EMA to explore the short-term associations among minority stress exposure and select mental health outcomes in SGM populations, indicating that on occasions where participants report heightened minority stress, they concurrently report heightened negative affect [22] and depression and anxiety symptoms [23,24]. However, very few studies involving SGMY have used EMA to link minority stress to suicidal ideation intensity. A recent exception is Mereish and colleagues’ daily diary study assessing associations between minority stress exposure and suicidal ideation intensity in SGMY over a 28-day period [25]. In a sample of 92 SGMY, the researchers found that on days when participants reported greater-than-usual exposure to distal and proximal minority stressors, they concomitantly experienced higher suicidal ideation intensity, emotional distress, and emotion dysregulation [25]. This research marks a significant advancement in SGMY suicidology, presenting the first within-person evidence illustrating the influence of daily minority stress exposure on fluctuations in one’s daily suicidal ideation intensity. Yet this study is limited in two ways: first, due to its daily diary design, the study was unable to explore shorter-term associations between minority stress exposure and suicidal ideation intensity, such as those occurring across hours; second, by recruiting participants exclusively from a single Northeastern city in the United States of America (US), the study may have been constrained in capturing the frequency of minority stress events reported, given the generally accepting public attitudes and policy protections for SGMY in this region [26].

To advance understanding of the role of minority stress in suicidal ideation intensity, the present study sought to develop and then assess feasibility and acceptability of an EMA study conducted via participants’ personal smartphones (i.e., “smartphone-based EMA study”) measuring minority stress and suicidal ideation intensity among SGMY at elevated risk of suicidality. In Study 1, we engaged in a multi-phase, iterative protocol development process, which included input from key community members. Prior research emphasizes the importance of involving community members, including members of the target population and experts, in EMA study development to improve acceptability, feasibility, safety, and compliance [19], particularly when exploring sensitive topics such as suicide [27–30]. In Study 2, we assessed feasibility and acceptability of the EMA study developed in Study 1 among 50 SGMY at elevated suicide risk residing in two states in the US Southeast, a geographic context with high structural stigma. Indeed, since 2023 in the US, over 1000 bills targeting the rights and visibility of SGM people have been introduced at the state level, at least 128 of which have been passed into law, mostly in Southeastern states [31,32]. This stigmatizing local context, marked by legislative constraints and political animus, offers an unfortunate yet opportune setting for investigating the real-time impact of minority stress on suicidal ideation intensity among SGMY at high risk.

## Study 1

The goal of Study 1 was to develop a smartphone-based EMA study capable of capturing experiences of minority stress and suicidal ideation intensity among SGMY residing in the US Southeast and at high risk of suicidality. The EMA study development process was guided by recommendations for developing an EMA protocol for studying the mental health impact of social media use in suicidal youth [28,33]. Study 1 involved a multi-phase, iterative process that involved obtaining input from relevant community members, including parents of SGMY, SGMY themselves, and experts (clinicians and researchers). All study procedures were approved by the Vanderbilt University Institutional Review Board.

## Methods

### Focus groups

To comprehensively inform an EMA study protocol designed to capture real-time minority stress exposure and suicidal ideation among SGMY at elevated suicide risk, we conducted focus groups with both parents of SGMY and SGMY themselves. Parent and SGMY focus groups were held separately, and recruitment was carried out independently for each group. As a result, participants in the parent focus groups and the SGMY focus groups were not recruited from the same households. To allow for greater geographic distribution of participants, focus groups were held virtually via Zoom.

Inclusion criteria for parent focus groups included being the legal guardian (i.e., parent or caregiver) of an SGMY ages 13–17-years-old, residing in Tennessee or a contiguous state, being fluent in English, and having access to a computer or smartphone capable of connecting to Zoom.

Inclusion criteria for SGMY focus groups included identifying as SGM (i.e., endorsing a non-heterosexual sexual orientation and/or having a sex assigned at birth that is incongruent with one’s current gender), being 13−24 years old, residing in Tennessee, being fluent in English, having access to both Zoom and a personal smartphone, reporting past-year suicidal ideation on the Ask Suicide***-***Screening Questions **(**ASQ) [34] tool, and reporting at least mild depression (score ≥5) on the Patient Health Questionnaire-9 (PHQ-9) [35]. Exclusion criteria for SGMY focus groups included being diagnosed with a psychotic disorder or reporting a suicide attempt within the previous 90 days. The inclusion and exclusion criteria for the SGMY focus groups were designed to align with those planned for the future EMA study, including limiting SGMY participants to those residing in the state of Tennessee. Excluding SGMY who had attempted suicide in the past 90 days reflects a level of risk that the study team could ethically manage, given that participants in the future EMA study would be geographically dispersed across the region and might not be connected to clinical care.

The recruitment period for parent and SGMY focus groups was February 18, 2022 to October 26, 2022. Recruitment for parent and SGMY focus groups involved multi-pronged strategies including outreach to child and adolescent behavioral health centers, distribution of recruitment materials to SGM-affirmative parent and youth organizations, and geo-targeted social media advertisements posted on Facebook, Instagram, and Twitter. All recruitment materials featured a QR code and a web link that directed potential participants to an online screening survey. Individuals who initially met the eligibility criteria per the online screener were then required to complete a brief screener call via Zoom with a trained research assistant (RA), whereby an RA explained the study’s procedures, confirmed eligibility, and obtained verbal consent and scheduling information. For eligible SGMY focus group participants under the age of 18, parental permission and youth assent were collected during this call. Focus groups were conducted over Zoom and followed separate semi-structured interview guides tailored to parents or SGMY, respectively. To ensure developmental similarities and comfort within SGMY focus groups, separate focus groups were conducted with 13–15-year-olds, 16–17-year-olds, and 18–24-year-olds. Both parent and youth focus groups consisted of small groups comprising 2 or 3 participants, with most involving 3 participants. Parents and youth were recruited and enrolled into focus groups separately; however, participants were not excluded from participation if their child or parent, respectively, had previously completed a focus group interview. While we are aware of at least 1 parent-youth dyad among the focus group interviewees, this was not assessed systematically and was not raised during interviews to maintain privacy of both parent and youth participants.

In both parent focus groups and SGMY focus groups, semi-structured interview guides included questions regarding exposure to minority stressors as well as participants’ opinions and preferences related to the development of a smartphone EMA study tailored to SGMY. Parents were asked their opinions regarding feasibility, privacy and confidentiality of EMA data, insights into developing and implementing a safety and risk monitoring protocol in the event of high-risk responses, and views on including SGM-specific features of the EMA study (e.g., pop-up messages with SGM-focused resources). A principal objective of parent focus groups was to elicit parents’ insights into study design features that would help to enroll SGM adolescents under age 18 given that parents are the primary gatekeepers for adolescent participation in research [36,37].

SGMY focus group participants were asked to reflect on factors such as timing and scheduling of EMA surveys, inclusivity of protocol language (e.g., using “LGBTQ+” versus “queer”), and components of the safety and risk monitoring protocol. SGMY focus group participants were also presented with EMA survey items as they were selected or developed and asked to provide their feedback and suggestions for refinements.

Parent and SGMY focus group participants were compensated with a $40 gift card after each focus group. All focus groups were conducted via Zoom and lasted approximately 60–90 minutes. Focus groups were audio recorded and transcribed using a HIPAA-compliant transcription company. Data from parent and SGMY focus groups were analyzed separately but followed the same analytic process. Dedoose software version 9.0.107 [38] was used to analyze the focus group data given its secure cloud-based portal where multiple users can engage in coding in real-time. Transcribed data were double-coded by two trained RAs (one graduate RA, one undergraduate RA) and analyzed using a thematic analysis approach [39]. First, RAs read and re-read all transcripts and jotted down initial notes to familiarize themselves with the data. Second, the RAs undertook an iterative coding approach to develop a preliminary codebook which was then refined and updated across several discussions among the research team. Once the codebook was finalized, the RAs used it to re-code the transcripts, which allowed for the application of newer codes to transcripts that were coded earlier in the process. Last, RAs developed preliminary themes that were finalized across a series of research team discussions.

Regarding positionality and reflexivity, our research team was comprised of a diverse group of clinicians, researchers, and undergraduate and graduate students holding varying social identities including sexual orientation, gender identity, race and ethnicity, class background, and educational attainment. In alignment with the principles of reflexive thematic analysis [39], regular team discussions were held to review coding and discuss key themes emerging from focus groups. To further support the research team in reflexivity during thematic analysis, the study’s clinical director, a licensed clinical psychologist, facilitated intermittent processing groups that provided a dedicated space for research staff, who primarily held SGM identities, to discuss the unique challenges and complexities they encountered while conducting qualitative, community-engaged research with SGMY and families in the US Southeast.

### Expert feedback

After parent and SGMY focus group feedback was integrated into a draft version of the EMA measures and protocol, this draft was reviewed through individual 45–60-minute meetings with six PhD-level experts in SGM mental health and EMA study design and implementation. Experts were selected based on their previous experience with EMA studies with suicidal youth and/or with SGM populations. Experts were asked to review the drafted EMA protocol and measures and provide feedback related to measurement of relevant constructs, EMA sampling protocol (e.g., timing, duration), and the safety and risk monitoring protocol. The first author conducted all interviews and took notes to capture the experts’ feedback. After each interview, the first author recorded memos reflecting on what was learned from the expert and how their feedback could be integrated into the drafted EMA protocol [40]. Expert feedback was discussed with the research team iteratively over several weekly meetings and utilized to refine the developing EMA protocol and measures.

## Results and discussion

### Parent focus groups

Parent focus groups were conducted with 16 parents (*M* = 47.44, *SD* = 4.41) of 17 SGMY ages 13–17 (*M* = 15.12, *SD* = 1.36). Among parent participants, 15 identified their sex assigned at birth as female, while one identified as male. Most parents were non-Hispanic White (**n* *= 15). Regarding gender identity, 14 identified their gender as woman and two identified as genderqueer or gender non-conforming. Parents described that their children held a diverse array of sexual orientations including pansexual (**n* *= 4), bisexual (**n* *= 3), asexual (**n* *= 3)**,** gay (**n* *= 2), lesbian (**n* *= 2), queer (**n* *= 2), and uncertain (**n* *= 1). Parents reported their SGMY children’s genders as girl (**n* *= 6), transgender boy (**n* *= 3), transgender girl (**n* *= 3), non-binary (**n* *= 3), boy (**n* *= 1), and genderfluid (**n* *= 1). During focus groups, parents identified numerous benefits and concerns regarding SGMY participation in a smartphone EMA study of minority stress and suicidal ideation. Table 1 provides an overview of parent-identified benefits and concerns, with salient quotes for each.

**Table 1:** Parent-identified benefits and concerns for SGMY participation in EMA studies.

	Domain	Description	Example quotes
**Parent-identified benefits**	Timely and necessary research in context	Parents emphasized that EMA research is needed, especially in the current sociopolitical climate, to better understand and address the mental health challenges faced by SGMY.	*“It’s very frustrating to be in this state...with how trans kids are being penalized in this state and not supported in this state. It’s bad enough with the rampant homophobia.”* *“I think we all know though, what the problem is. It’s not the kids. It’s the environment.”*
	Informing mental health resources	Parents recognized that involving SGMY in EMA research could help to inform the development of needed mental health resources that are tailored to their unique needs.	*“Well, there’s not a lot of resources around here. You [parents] got to make them happen, be more involved.”*
	Filling research gaps	Some parents expressed frustration over the lack of existing research in this area.	*“And so, I’m also always really frustrated with the fact that there’s not a lot of research that’s been done in these areas… it’s important and it obviously matters.”*
**Parent-identified concerns**	Research intentions	Parents expressed apprehension about how real-time data might be used by researchers and other people with nefarious intent.	*“I think my biggest concern was, A, is this, I mean, even though it’s confidential, is it-is it a supportive space for LGBTQ [youth], or is any of this information going to be used in a way, obviously, [apprehensively chuckles] that would be harmful to our kids?”*
	Privacy and confidentiality	Participants were concerned about privacy and confidentiality of the real-time information provided by SGMY during the EMA research, particularly considering sensitive topics including minority stress and suicide risk.	*“I think honestly, they’re [parents of SGMY] really concerned about their privacy…being able to be tracked somehow some way... That in and of itself is like even more, I would say, on their radar of why they would maybe be opposed to having any kind of location tracker into [the EMA app].”*
	Safety for SGMY	Specific concerns were raised about potential risks for SGMY, such as the possibility of someone noticing an SGM survey notification or safety implications related to GPS tracking or monitoring features built into many EMA apps.	*“I think also a safety feature on that would be that the push notification would not identify it as an LGBTQ survey because they would - if they were in a situation where that wasn’t a safe thing, I would not want that flashing up on their phone.”*
	Sociopolitical climate	Participants were wary of potential legal ramifications or consequences of EMA research participation, such as involvement with child protective services. Parents also expressed concerns about the impact of a potential study data leak within the current sociopolitical climate.	*“And that’s the reason that I think, uh, most of the people that I talk to were like, “Mm-mm [no], I’m not doing study right now. [laughs] ‘cause like, ‘I don’t know who’s gonna get that information.’” And if that’s gonna eventually mean a knock on the door from [Department of Children’s Services], you know?”* *“I worry that they’re going to go the route that Texas has gone where I could be accused of abusing my child for using his pronouns…”*

EMA = ecological momentary assessment; GPS = global positioning system

Parent feedback was integrated into developing both the EMA study protocol and participant-facing materials. For example, our study team initially considered passively assessing SGMY participants’ real-time locations through global positioning system (GPS) monitoring built-in to the smartphone EMA application (“app”). However, after parents expressed concerns about this data collection approach during focus group interviews, GPS monitoring was removed from the EMA study protocol. Additionally, due to parents’ concerns about SGMY privacy if they were completing the EMA surveys in a public place where someone could see their smartphone, our team revised pop-up reminder notifications to use general language and not mention the SGM focus of the study. Further, due to parents’ concerns about privacy and confidentiality of real-time data collected from SGMY within the current hostile sociopolitical climate in the US Southeast, we carefully revised consent language to clearly document how data would be stored securely. Further, we developed a visually appealing information sheet for parents and SGMY titled, “Who We Are, What We Do, and How We Keep Your Information Safe” that describes the EMA study data security processes in detail. Because this EMA study was funded by the National Institutes of Health (NIH), we also included information about the NIH Certificate of Confidentiality further protecting research participant privacy. Last, based on parent feedback about strategies to promote engagement with SGMY and their families in the US Southeast, our team developed a public-facing website with study staff biographies and photographs to enhance transparency and trust between potential SGMY participants, their parents, and the study team.

### SGMY focus groups

SGMY focus groups consisted of sixteen participants ages 14–24 years old (**M* *= 18.38, *SD* = 2.66) who resided in Tennessee. Among these participants, eight were youth (range = 14–17 years old) and eight were young adults (range = 19–24 years old). Sexual orientation of SGMY focus group participants included bisexual (**n* *= 7), lesbian (**n* *= 2), pansexual (**n* *= 2), queer (**n* *= 2), gay (**n* *= 1), aromantic bisexual (**n* *= 1), and panromantic asexual (**n* *= 1). All SGMY participants were assigned female sex at birth, but endorsed a relatively diverse array of gender identities including girl/woman (**n* *= 6), boy/man (**n* *= 4), genderqueer (**n* *= 2), trans girl/woman (**n* *= 1), trans boy/man (**n* *= 1), genderfluid (**n* *= 1), and Two-Spirit (**n* *= 1).

SGMY focus groups identified four primary themes related to EMA study development, including EMA measure refinement, logistical considerations, parental consent considerations, and safety and risk monitoring protocol considerations, each described in greater detail below.

#### EMA measure refinement.

During focus groups, SGMY were presented with potential EMA survey items and asked to share their perspectives on wording and content. Participants provided valuable feedback for altering survey questions asking about social and place-based context, social media use, and minority stress. Participant feedback was also used to develop new EMA questions. For instance, SGMY participants highlighted that seeing and reading negative news related to SGM people was a near-continual source of minority stress and was highly prevalent within the hostile sociopolitical climate in the US Southeast. Thus, we added two questions to the set of EMA measures: one capturing recent exposure to negative news media (e.g., a social media post, a news headline) and a follow-up question regarding whether or not the negative news media was related to LGBTQ+ people or communities.

#### Logistical considerations.

SGMY focus group participants provided detailed logistical considerations for engaging in a smartphone EMA study including feedback related to compensation incentive structures, pop-up messages and resources, and reminder notifications. Participants expressed a common sentiment that SGMY motivation to complete EMA surveys could be enhanced through cash incentives and bonuses for completing a certain number of surveys per week. Additionally, strategies like pop-up messages featuring both local and national SGM-resonant mental health and community resources were suggested to maintain motivation over the 28-day period.

SGMY focus group participants also provided helpful insight into the ideal number of EMA surveys to administer per day. Participants shared that 3 or 4 daily surveys would be enough to capture the breadth of their daily experiences without feeling overwhelming or interfering greatly with their school and work schedules, with one participant remarking: “I feel like...six surveys a day…I feel that would be too much, especially when teens are in school and sometimes they can’t answer them. I feel like three a day gives them enough time to experience more things, to put more into the diary or whatever at the end of the surveys at the end of the day... If you have three, more happens between them so you have more impact on the results”.

Relatedly, focus group participants described a range of school policies regarding mobile phone use that they could foresee as potential barriers to survey completion. Some reported that phone access was entirely restricted during the school day, while others attended schools with more flexible policies or had free periods that could accommodate survey completion. When asked about the ideal number of daily surveys (e.g., 2, 3, 4, or 6) considering phone access at school, participants generally agreed that 3 surveys per day would best fit into students’ schedules. As one participant explained, “I feel like it would work out best if it was the three [surveys per day]. They had one in the morning before they get ready for school or one during lunch if they’re allowed to have their phones…”

Regarding communication preferences, participants indicated a preference for text messaging rather than email or phone calls when interacting with study staff. SGMY also emphasized the importance of frequent reminder notifications to prompt EMA survey completion. This perspective was particularly relevant for participants managing busy school schedules and extracurricular activities and work commitments, as well as for those experiencing neurodiversity. Notably, during screening, 43.8% of focus group participants reported that they had been diagnosed with Attention-Deficit/Hyperactivity Disorder (ADHD) and 6.3% reported being diagnosed with an Autism spectrum disorder. Related to logistical considerations for receiving reminders to complete EMA surveys in the context of neurodiversity, one participant shared, “Because I have really bad ADHD and I can’t even answer texts sometimes, maybe [receiving a reminder notification every] five minutes would be a bit excessive, but if it was something even like 15 or maybe even 30 [minutes] if it’s over a two-hour period, I guess, I think that could be very helpful.” Based on this feedback, we developed a reminder notification schedule for the EMA study. After the participant received the first notification to complete the EMA survey, the app deployed up to six reminder notifications spaced approximately 15 minutes apart over a two-hour period until the survey disappeared.

#### Parental consent considerations.

SGMY participants frequently voiced concerns about requiring parent permission for SGMY under the age of 18 to participate in an EMA study focused on minority stress and suicidal ideation intensity. Their apprehensions centered on the potential impact of parent permission requirements on SGMY participant safety, especially for those with non-affirming parents. Additionally, participants expressed concerns about how these requirements might influence the willingness of SGMY under the age of 18 to participate in the EMA study as well as how requiring parent permission, particularly in the context of the US Southeast where SGMY frequently face family rejection [41], could introduce sampling bias. Based on this feedback, we planned to seek a waiver of parental permission from the IRB for the EMA study (however, based on institutional policy, this was ultimately not possible; see *Challenges and Opportunities for Future EMA Research with SGMY* in Overall Discussion section).

#### Safety and risk monitoring protocol considerations.

SGMY focus groups participants provided feedback related to the safety and risk monitoring protocol. One major concern involved the logistics of enlisting an emergency contact, particularly parents, with many participants seeking to understand when and under what circumstances the research team would reach out to their emergency contacts. Participants also inquired about the threshold for triggering the initiation of the safety and risk monitoring protocol. Many participants shared negative past experiences related to risk intervention responses, which contributed to a sense of wariness among participants about potentially involving emergency services in the event of a crisis situation.

In response to this feedback, we refined several aspects of the protocol (see *Safety and Risk Monitoring Protocol* in Study 2 for full details). First, due to SGMY requesting clarity on when an emergency contact would be engaged, we edited the protocol to clarify that the SGMY participant is the first point of contact for any risk assessment outreach from study staff. If an SGMY participant does not answer this risk assessment phone call, they are then sent clear timeframes by voicemails and text messages, informing them that if they do not respond within two hours, the study team will contact their emergency contact. Second, based on SGMY focus group feedback, the risk monitoring protocol was amended so that the involvement of emergency services was considered only in cases of high risk where a participant’s emergency contact was unresponsive. Rather than contacting 911 or involving law enforcement, our team instead compiled a list of mobile crisis services available in each participant’s local area that would be contacted if emergency services were needed. Third, to ensure participants were aware of all safety and risk monitoring protocol procedures, we provided comprehensive information about the protocol during the consent (and assent, as applicable) processes.

Based on parent and SGMY focus groups, as well as a literature review of existing EMA studies, Table 2 provides an overview of the drafted EMA measures and protocol and the primary sources of input on the respective topic.

**Table 2:** Drafted EMA measures and protocol and sources of input.

		Parent focus groups	SGMY focus groups	Literature review
**EMA survey measures**				
**Context**	Social context (e.g., who are you with?)		X	X
	Place-based context (e.g., where are you?)		X	X
	Social safety (e.g., how accepting of your LGBTQ+ identity is this place?)	X	X	
**Suicidal ideation and its precursors**	Sleep			X
	Positive and negative affect			X
	Interpersonal needs (e.g., burdensomeness)			X
	Emotion regulation			X
	Suicidal ideation			X
**LGBTQ+ identity considerations**	Distal minority stress (e.g., discrimination)	X	X	X
	Proximal minority stress (e.g., internalized stigma)		X	X
	Negative news and media messaging	X	X	
	LGBTQ+ community connectedness	X	X	
**Interpersonal factors**	Online and in-person interactions		X	X
	Relationships (e.g., peer, family, partner)	X	X	X
**Qualitative/ diary feedback**	Positive and negative events			X
**Protocol and study design**		**Parent focus groups**	**SGMY focus groups**	**Literature review**
	Schedule/timing of EMA surveys	X	X	X
	Number of EMA surveys per day		X	X
	Duration of study (i.e., number of days)		X	X
	Deployment method (e.g., app versus text)		X	
	Safety and risk monitoring protocol	X	X	X
	Pop-up messages and resources		X	
	Informed consent processes and considerations	X	X	X
	Incentive structure		X	X
	LGBTQ+ specific protocol considerations	X	X	

### Expert feedback

Six PhD-level experts provided feedback on the drafted EMA measures and protocol. With regards to EMA measures, experts recommended removing extraneous items to minimize participant burden and enhance compliance. For instance, the initially drafted measures included the Patient Health Questionnaire-2 (PHQ-2) [42] and General Anxiety Disorder-2 (GAD-2) [43] to assess depressive and anxiety symptoms. However, experts suggested that these were duplicative to mood assessments already part of the Profile of Mood States (POMS) [44] and should be excluded. Similar feedback was provided regarding items assessing suicidal ideation intensity. One expert in EMA studies with adolescents recommended calculating objective readability assessments of EMA measures to ensure comprehension across participants of varying ages or grade levels, a suggestion which we heeded, as reported next.

Experts unanimously agreed that administering three EMA surveys per day over 28 consecutive days constituted an ideal assessment schedule for the constructs of interest. One expert, with extensive experience using EMA to assess minority stress, emphasized that a 28-day duration, as compared to shorter study periods, would likely provide sufficient variability for analysis, particularly given the relatively infrequent occurrence of minority stress events (e.g., acute experiences of anti-SGM harassment) on an hourly or even daily basis. Another expert who specializes in the use of EMA to measure suicidal ideation intensity, noted that three daily surveys strike an appropriate balance such that they are frequent enough to capture meaningful within-person fluctuations in symptoms over time, while remaining feasible in terms of the research team’s capacity to monitor and respond to participant risk. Experts additionally suggested effective advertising and recruitment strategies tailored to attract and retain SGMY, safety and risk monitoring protocol considerations, and considerations for incentives and bonus structures. Furthermore, two experts proposed incorporating an optional exit interview component for participants enrolled into the EMA study to gather valuable insights for informing future studies as well as the development of interventions. We integrated these suggestions into our final EMA study protocol.

### Objective readability assessment

We evaluated the objective readability of the refined EMA measures by calculating the Flesch Readability Ease and Flesch-Kincaid Grade Level scores [45]. The Flesch Readability Ease score was determined to be 64.6. Scores falling between 60–70 are categorized as “Plain English” and should be easily understood by individuals aged 13 and older. The Flesch-Kincaid Grade Level was computed at 5.9, indicating that the EMA measures have an objective readability equivalent to approximately the sixth-grade reading level. These results suggest that the EMA measures should be easily readable for all potential participants.

### Finalized EMA study protocol: Project SPIRiT (Suicide Prediction in Real-Time)

**Fig 1:**
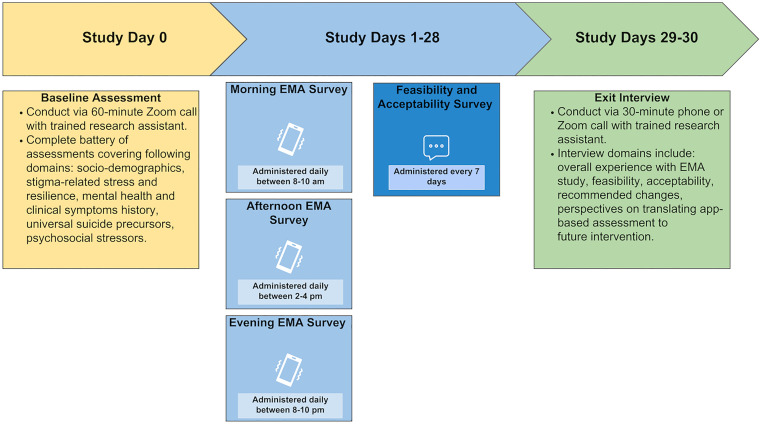
Overview of EMA study protocol and assessment schedule.

Through a multi-phase process based on recommendations for rigorously designing EMA studies [28,33], we developed Project SPIRiT, a smartphone-based EMA study targeting SGMY aged 13–24-years-old in the US Southeast and at elevated suicide risk. The overarching goal of Project SPIRiT is to elucidate within-person associations among minority stress exposure and suicidal ideation intensity across a short (hours) timescale and within everyday life. Project SPIRiT involves enrolling participants into an intensive longitudinal study protocol involving a baseline assessment, 28-day EMA study, weekly feasibility and acceptability surveys, and an optional exit interview. Study procedures are detailed in-depth below in Study 2. Fig 1 provides an overview of the Project SPIRiT study protocol that was developed in Study 1.

## Study 2

The goal of Study 2 was to implement the Project SPIRiT study protocol in a sample of SGMY in the US Southeast and assess the feasibility and acceptability of the protocol using compliance metrics (e.g., % of EMA surveys completed) as well as quantitative and qualitative data from weekly feedback surveys and exit interviews.

## Method

### Participants

Participants were included in the study if they: were 13–24 years old, identified as SGM based on self-reported sexual orientation and/or gender identity, reported past-year suicidal ideation on the ASQ [34], reported at least mild depression on the PHQ-9 [35], and had access to a personal smartphone with iOS or Android operating systems, both of which are compatible with the EMA software (MetricWire). Initially, participants were required to reside in Tennessee, but after several months of slower recruitment of adolescents (13–17 years old) in Tennessee, this criterion was expanded to include residents of Alabama, which is contiguous to Tennessee and shares similarities with regard to sociopolitical climate, urban-rural divide, and proportion of youth (<18) residents [46]. Participants were excluded if they: did not meet inclusion criteria, were unable or unwilling to provide informed consent (for participants aged 18–24) or parent permission and adolescent assent (for participants aged 13–17), had been diagnosed with a psychotic disorder, or had attempted suicide within the past 90 days. The recruitment period for Project SPIRiT was March 30, 2023 to June 20, 2024.

**Fig 2:**
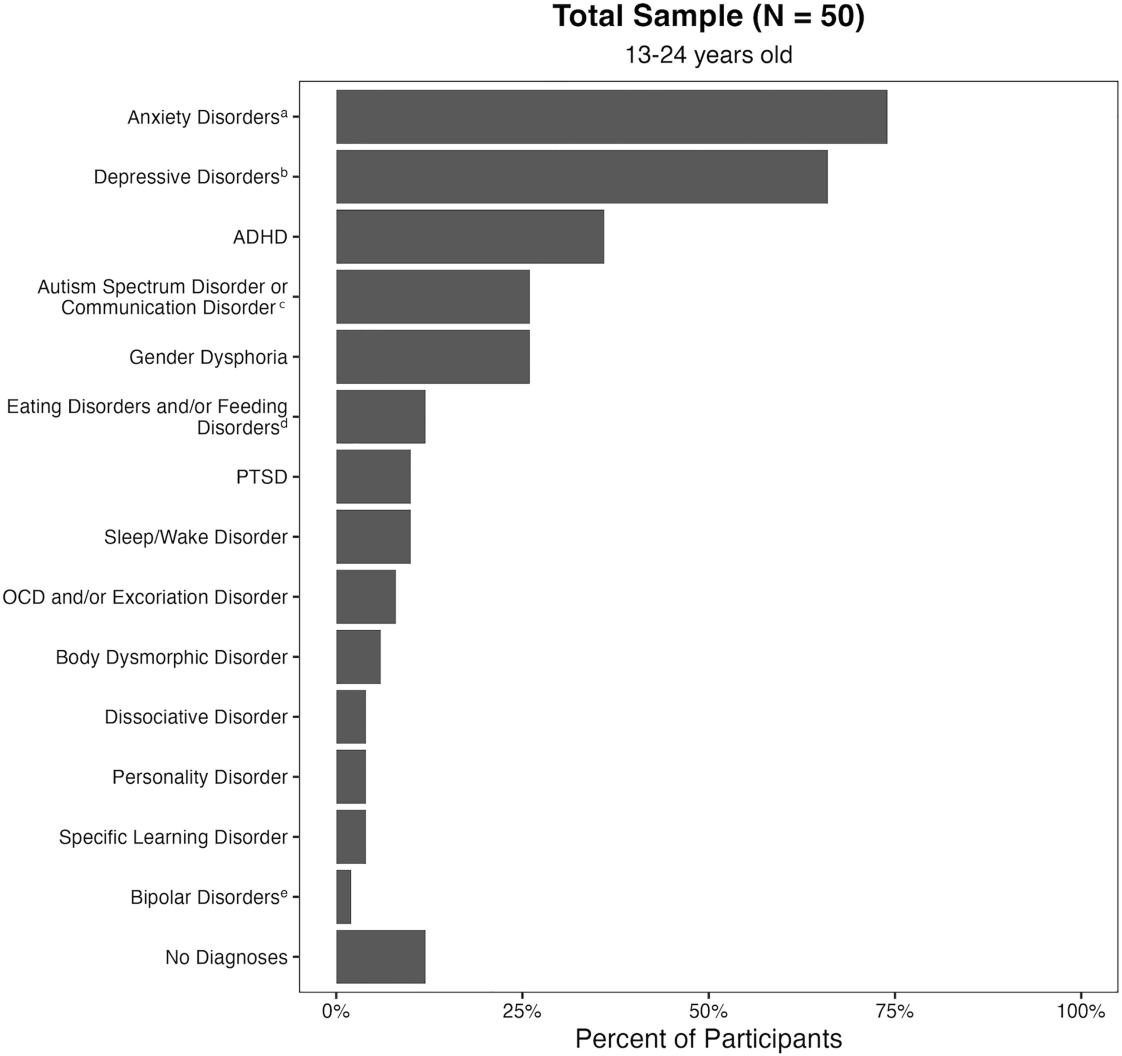
Self-reported clinician-diagnosed mental disorders at baseline assessment. ADHD = attention-deficit/hyperactivity disorder; PTSD = post-traumatic stress disorder; OCD = obsessive-compulsive disorder. ^a^ Anxiety Disorders includes participants who selected at least one of the following: Generalized Anxiety Disorder, Social Anxiety Disorder, Anxiety (Unspecified), Agoraphobia, Separation Anxiety Disorder, Panic Disorder, and/or Selective Mutism. ^b^ Depressive Disorders includes participants who selected at least one of the following: Depression (Unspecified), Major Depressive Disorder, Mood Disorder (Unspecified), Disruptive Mood Dysregulation Disorder, and/or Premenstrual Dysphoric Disorder. ^c^ Autism Spectrum Disorder or Communication Disorder includes participants who selected Autism Spectrum Disorder and/or Asperger’s Disorder, or Communication Disorder. ^d^ Eating Disorders and/or Feeding Disorders includes participants who selected at least one of the following: Anorexia Nervosa, Binge Eating Disorder, Bulimia Nervosa, and/or Feeding Disorder. ^e^ Bipolar Disorders includes a participant who reported Hypomania.

The final sample included 50 SGMY (*M*_*age*_ = 18.38, *SD *= 3.02; range = 13–24). In total 19 (38.0%) participants were 13–17 years old and 31 (62.0%) were 18–24 years old. Table 3 provides sample sociodemographic and clinical severity data from participants’ baseline assessment. In total, 38 (76.0%) participants reported a female sex at birth, 11 (22.0%) reported a male sex at birth, and 1 (2.0%) reported having been assigned intersex. The most prevalent gender identities endorsed were girl/woman (*n* = 17, 34.0%), non-binary (*n* = 14, 28.0%), and trans boy/man (**n* *= 11, 22.0%). The most prevalent sexual orientations endorsed were bisexual (**n* *= 20, 40.0%), queer (**n* *= 12, 24.0%), and lesbian (**n* *= 8, 16.0%). On a question asking participants to classify their gender modality, 29 (58.0%) identified as transgender or gender diverse. Regarding history of suicidal thoughts and behaviors, 35 (70.0%) had seriously considered suicide, 35 (70.0%) had engaged in non-suicidal self-injury, and 17 (34.0%) had attempted suicide. Among those who had made a previous suicide attempt, on average participants had made 2.65 attempts (*SD *= 1.32) with a median of 2 attempts. Fig 2 depicts the prevalence of participants’ self-reported lifetime mental disorders at baseline, indicating a high prevalence of mental disorders; approximately three-quarters reported an anxiety or depressive disorder diagnosis and approximately one-quarter reported an ADHD, autism or communication disorder, or gender dysphoria diagnosis.

**Table 3:** Sample sociodemographic characteristics and clinical severity data.

	Variable	Total (*N *= 50)	Young adults (*n *= 31)	Adolescents (*n *= 19)
		*M* [*SD*]/ *n* (%)	*M* [*SD*]/ *n* (%)	*M* [*SD*]/ *n* (%)
**Sociodemographic Characteristics**	**Age**	18.52 [3.02]	20.45 [1.89]	15.37 [1.38]
	**Race** ^a^			
	Asian	2 (4.0)	2 (6.5)	0 (0.0)
	Black/African American	4 (8.0)	4 (12.9)	0 (0.0)
	White	41 (82.0)	24 (77.4)	17 (89.5)
	Multiple Races	2 (4.0)	0 (0.0)	2 (10.5)
	Another race	1 (2.0)	1 (3.2)	0 (0.0)
	**Hispanic or Latinx**	4 (8.0)	3 (9.7)	1 (5.3)
	**Non-Hispanic White**	39 (78.0)	22 (71.0)	17 (89.5)
	**Sex Assigned at Birth**			
	Female	38 (76.0)	22 (71.0)	16 (84.2)
	Male	11 (22.0)	8 (25.8)	3 (15.8)
	Intersex	1 (2.0)	1 (3.2)	0 (0.0)
	**Binary or Nonbinary Gender** ^b^			
	Binary	28 (56.0)	19 (61.3)	9 (47.4)
	Nonbinary	21 (42.0)	11 (35.5)	10 (52.6)
	Neither	1 (2.0)	1 (3.2)	0 (0.0)
	**Gender Modality** ^c^			
	Transgender	28 (56.0)	16 (51.6)	12 (63.2)
	Cisgender	21 (42.0)	15 (48.4)	6 (31.6)
	Neither	1 (2.0)	0 (0.0)	1 (5.3)
	**Gender Identity** ^d^			
	Girl or Woman	17 (34.0)	11 (35.5)	6 (31.6)
	Boy or Man	6 (12.0)	6 (19.4)	0 (0.0)
	Trans Girl or Woman	4 (8.0)	3 (9.7)	1 (5.3)
	Trans Boy or Man	11 (22.0)	6 (19.4)	5 (26.3)
	Nonbinary	14 (28.0)	9 (29.0)	5 (26.3)
	Gender Fluid	2 (4.0)	2 (6.5)	0 (0.0)
	Gender Nonconforming	2 (4.0)	1 (3.2)	1 (5.3)
	Genderqueer	3 (6.0)	1 (3.2)	2 (10.5)
	Agender	4 (8.0)	3 (9.7)	1 (5.3)
	Another gender identity	4 (8.0)	3 (9.7)	1 (5.3)
	**Sexual Orientation** ^d^			
	Straight	1 (2.0)	1 (3.2)	0 (0.0)
	Lesbian	8 (16.0)	6 (19.4)	2 (10.5)
	Gay	6 (12.0)	4 (12.9)	2 (10.5)
	Bisexual	20 (40.0)	13 (41.9)	7 (36.8)
	Pansexual	5 (10.0)	4 (12.9)	1 (5.3)
	Queer	12 (24.0)	10 (32.3)	2 (10.5)
	Questioning	6 (12.0)	2 (6.5)	4 (21.1)
	Asexual	7 (14.0)	5 (16.1)	2 (10.5)
	Another sexual orientation	1 (2.0)	0 (0.0)	1 (5.3)
**Clinical Severity**	**Lifetime Suicide Attempt**	17 (34.0)	13 (41.9)	4 (21.1)
	**Number of Lifetime Suicide Attempts** ^e^	2.65 [1.32]	3.00 [1.29]	1.50 [0.58]
	**Age (in years) of First Suicide Attempt** ^e^	13.06 [2.93]	13.31 [3.30]	12.25 [0.96]
	**Years Since Last Suicide Attempt** ^e^	2.94 [2.11]	3.15 [2.34]	2.25 [0.96]
	**Lifetime Serious Suicide Ideation**	35 (70.0)	22 (71.0)	13 (68.4)
	**Lifetime Non-Suicidal Self-Injury**	35 (70.0)	20 (64.5)	15 (78.9)
	**Age (in years) of First Non-Suicidal Self-Injury** ^f^	12.54 [2.98]	13.50 [2.76]	11.27 [2.87]
	**Years Since Last Non-Suicidal Self-Injury** ^f^	0.83 [1.38]	1.15 [1.63]	0.40 [0.83]
	**Lifetime Mental Health Treatment**	46 (92.0)	27 (87.1)	19 (100.0)
	**Current Mental Health Treatment**	31 (62.0)	16 (51.6)	15 (78.9)

^a^The multiple races option includes a participant who selected Asian, White, and More than one race, as well as a participant who selected White and More than one race. The Other category includes a participant who self-identified Race as Latinx.

^b^Binary or nonbinary gender was assessed with the following question: “When we describe who participated in our study, which of the categories below would you like us to include you in?” Participants could select one of the four options: a) Binary (someone who identifies as completely a man/male or woman/female), b) Nonbinary (someone who has an identity other than completely woman/female or man/male), c) Neither binary nor nonbinary describe me because [text response], d) Unsure because [text response].

^c^Gender modality was measured through the following question: “When we describe who participated in our study, which of these categories would you like us to include you in?”. Participants could endorse one of the four options: a) A trans/transgender category (usually refers to people who were given a gender and/or sex label at birth that does not accurately represent them), b) A cisgender category (refers to people who are the same gender and/or sex they were assigned at birth), c) Neither cisgender nor transgender describe me because [text response], d) Unsure because [text response].

^d^Percentages exceed 100% for Gender Identity and Sexual Orientation since participants could select multiple choices.

^e^This variable includes responses from 17 participants who had indicated a history of suicide attempt.

^f^This variable includes responses from 35 participants who had indicated a history of non-suicidal self-injury.

### Measures and procedure

All study procedures were approved by the Vanderbilt University Institutional Review Board. Participants were recruited through various strategies, including outreach to SGMY-serving organizations and events, flyers placed in libraries, pediatricians’ offices, outpatient mental and behavioral health clinics, and paid and unpaid geo-targeted social media advertisements posted on Instagram and Facebook.

Study recruitment materials included a clickable link or QR code that directed potential participants, or their legal guardians for those aged 13–17, to a brief screening survey to determine preliminary interest and eligibility and collect contact information. Interested participants then completed a Zoom screening call with an RA. For participants aged 13–17, a legal guardian (in all cases, a parent) joined the call. During this call, eligibility information was confirmed by assessing all inclusion criteria, additional details about study procedures were provided, and informed consent was obtained from participants aged 18–24 and parent permission and adolescent assent from those aged 13–17. Participants were then scheduled for their baseline assessment. The study consisted of four components: the baseline assessment, a 28-day EMA study, weekly feasibility and acceptability surveys, and an exit interview (see Fig 1 for study flow).

#### Baseline assessment.

The baseline assessment was conducted via Zoom by a Masters-level RA and took approximately 60−90 minutes to complete. The baseline assessment included three components: having the SGMY participant complete a battery of self-report surveys, completing a Stanley-Brown Safety Plan [47] to be used in the event of a high-risk flag (see *Safety and Risk Monitoring Protocol*), and helping the participant download the EMA smartphone app along with training on the EMA software and study protocol. Constructs assessed in the baseline battery of self-report measures covered five domains: (1) demographics, (2) distal and proximal minority stressors (e.g., Gender Minority Stress and Resilience Scale for Adolescents) [48], (3) clinical severity history (e.g., Suicide Ideation Attributes Scale) [49], (4) universal suicide risk factors (e.g., Interpersonal Needs Questionnaire-15) [50], and (5) psychosocial stress and resilience factors (e.g., Multidimensional Scale of Perceived Social Support) [51]. All baseline assessments and their sources are outlined in S1 Table in the Supporting Information.

#### 28-day EMA measures.

Each day, participants completed three EMA surveys through MetricWire, a HIPAA-compliant smartphone software developed for EMA survey management. EMA surveys were signal-contingent (i.e., initiated by the software during pre-scheduled times) and deployed within pre-specified time blocks in participants’ local time zones (morning: 8–10 am; afternoon: 2–4 pm; evening: 8–10 pm). Participants had up to 2 hours to complete the EMA survey before it disappeared. During that window, participants received up to 6 reminder notifications at the following intervals: 5 mins, 15 mins, 30 mins, 45 mins, 60 mins, and 90 mins. Each morning before 10am in the researchers’ local time zone, RAs checked each participant’s EMA survey responses from the previous day. Participants who did not complete any of the previous day’s three EMA surveys were sent a personalized text from an RA asking if there were any technical issues the study team could assist with. The median completion time across all EMA surveys was 167 seconds or 2.78 minutes.

All EMA surveys included a core block of 42 EMA questions capturing real-time social and place-based context, minority stress, interpersonal conflict, positive and negative online and in-person interactions, mood, suicidal ideation intensity, and other relevant time-varying psychosocial constructs (e.g., belongingness). The morning survey included an additional 2 items assessing the duration and quality of the participant’s sleep. The evening survey included an additional 6 items asking participants to rate their level of hopefulness for the next day as well as 2 open-ended question asking participants to describe their most positive and negative events of the day followed by a checklist of emotion regulation strategies that they used in response to their most negative event. All EMA survey items and their sources are outlined in S2 Table in the Supporting Information.

#### Weekly feasibility and acceptability surveys.

After each week of participation, participants received a text message with a link to a brief feasibility and acceptability survey programmed on REDCap survey software that asked participants to report on their experiences with the EMA study. Using items with Likert scales from 0 (*not at all*) to 10 (*very much*), these weekly surveys assessed: i) participants’ privacy, ii) the protocol’s ability to capture participants’ feelings, and whether questions were iii) easy to understand, iv) annoying/disruptive, v) boring, and vi) interesting/engaging. In addition, participants indicated via a yes-or-no question whether they had experienced any technical difficulties with the EMA survey in the previous week and described the technical difficulty.

#### Exit interview.

All participants were offered the option to complete an exit interview, conducted by phone or Zoom with an RA, where they could provide feedback on their study experience. Exit interviews probed participants’ perspectives on the following topics: likes and dislikes of completing the EMA surveys, any difficulties completing the surveys, engagement in and/or repetitiveness of the process, whether and how the EMA surveys had affected their feelings and emotions, feedback on communication with the research team, and potential intervention implications. Questions regarding engagement with the research team and potential intervention implications were added iteratively to the semi-structured exit interview guide based on qualitative data from participants gathered during initial exit interviews.

#### Compensation structure.

The study compensation structure was designed to balance increasing participant motivation while avoiding potential coercion and was based on feedback from Study 1 focus groups with parents and SGMY. Participants received $40 for completing the baseline assessment, $1 for each completed EMA survey, with an additional $10 weekly bonus if they complete at least 70% of the surveys within the previous 7 days. An extra $10 was offered for completing the optional exit interview. In total, participants could earn up to $186 throughout the entire study period.

### Safety and risk monitoring protocol

Based on feedback from parents, SGMY, and experts in Study 1, and in consultation with previous research [14] and the clinical members of our research team, we developed a structured safety and risk monitoring protocol to ensure participant safety during the 28-day study period. At the start of each EMA survey, participants viewed a message reminding them that their responses were not monitored in real-time and that they would be asked questions about their suicidal ideation intensity. At the end of each EMA survey, all participants received a message with online crisis resources, including links to Crisis Text Line, NowMattersNow, the 988 suicide crisis hotline, and The Trevor Project. The message also reminded participants to review their personalized Stanley-Brown Safety Plan completed during the baseline assessment.

#### Risk assessment trigger.

The safety and risk monitoring protocol was triggered by the participants’ responses to an item assessing their active suicidal ideation (i.e., Right now, what is your urge to kill yourself?). Responses were categorized into three risk levels based on the Likert-type response options from 0 (*not at all*) to 10 (*extremely*): None/Low (0–5), Moderate (6–8), and High (9–10). For None/Low Risk (0–5) responses, participants did not receive any immediate intervention except for the standard crisis resource list provided at the end of each EMA survey. For Moderate Risk (6–8) responses, participants were presented with the same crisis resources along with an additional message emphasizing the importance of seeking help from a therapist or supportive friend or family member.

For High Risk (9–10) responses, participants received an immediate pop-up message advising them to contact support services and informing them that study staff would follow up via phone within 12 hours. This response also triggered an automated email and text message to all clinical study staff (i.e., PI, study clinical director, and Master’s-level RAs) with the participant’s ID number. If participants could not be contacted via phone within two hours of outreach, their emergency contact was notified. During the risk assessment phone call with the participant, a Master’s-level RA conducted a brief risk assessment using an adapted screen version of the Columbia-Suicide Severity Rating Scale (C-SSRS) [52] to determine the participant’s risk level and necessary immediate actions. Based on the participant’s responses to the C-SSRS, actions ranged from reminding participants of their Stanley-Brown Safety Plan and encouraging connection with mental health services to more urgent interventions, such as obtaining commitment from the participant to go to the ER, facilitating a three-way call with a parent (for adolescents) or other emergency contact (for young adults) to accompany the participant to the ER, or initiating a three-way call with emergency services (i.e., local mobile crisis unit).

#### Manually checking negative daily diary responses.

Every morning, RAs reviewed diary responses submitted by participants on the previous evening’s survey where they described their most negative event of the day. In consultation with the study’s clinical director, a licensed clinical psychologist, RAs reviewed the responses for any description of self-harm including suicide attempt. In the event of written disclosure of self-harm or a suicide attempt in the diary entry, the High Risk protocol described previously was triggered.

#### Hospitalization.

In the case of psychiatric hospitalization during the 28-day study period, participants were able to pause participation and re-start EMA surveys after the hospitalization upon re-screening to assess inclusion and exclusion criteria and to re-consent.

### Data analysis

#### Feasibility.

Feasibility was assessed through participants’ EMA survey compliance over the 28-day study period. Compliance was assessed descriptively (i.e., overall, week-to-week, by survey type) and as a function of age group (young adults vs. adolescents) and baseline clinical severity (presence of suicide attempt history, presence of non-suicidal self-injury history) to understand factors that may enhance or reduce compliance rate. We used a combination of marginal means analyses and multilevel modeling. Statistical analyses and visualizations were conducted using SPSS version 28.0.1.1 and R version 4.3.1 with statistical significance assessed at α < .05.

#### Acceptability.

Acceptability was assessed using both weekly feedback surveys and the optional qualitative exit interviews. We investigated acceptability using descriptive statistics and assessed differences between age groups (young adults or adolescents). For analyses of marginal means, responses were averaged for each participant across the weeks of their participation prior to computing descriptive statistics and inferential analyses, as there was a small amount of missingness in responses to weekly feedback surveys (*n*_*Week* *1*_ = 49/50, *n*_*Week 2*_ = 43/50, *n*_*Week 3*_ = 43/50, *n*_*Week 4*_ = 46/50; all participants responded to at least one survey). To account for the hierarchical structure of the data, we also estimated multilevel models with random intercepts and time (operationalized as a continuous variable denoting week of response, ranging from 0 to 3) as a predictor of each variable that was assessed in the weekly feedback surveys.

Exit interviews were conducted via Zoom, lasted approximately 20 minutes, and were audio recorded and transcribed using a HIPAA-compliant transcription company. Transcribed data were double-coded by two trained RAs (one graduate RA, one undergraduate RA) and analyzed using qualitative content analysis (QCA) [53,54]. The exit interviews, while semi-structured, followed a consistent interview guide designed to elicit responses on a standard set of topics (e.g., experiences of survey scheduling, burden, duration, overall feasibility and acceptability). Given the structured nature of these interviews and our goal of capturing the relative salience of specific feedback topics across participants, QCA was selected as the optimal qualitative approach. To begin the QCA process, RAs reviewed all of the data and completed marginal note-taking. RAs met to create an initial coding frame, which was refined after feedback from the research team. This revised frame was tested, evaluated, and then modified as needed. The final coding frame was then applied to the entire dataset. After applying the coding frame to the entire dataset, frequencies were quantified by dividing the number of participants who endorsed a theme over the total number of participants and salient quotes were extracted.

## Results and discussion

### Feasibility

#### EMA compliance.

In total, the 50 SGMY participants completed 3,369 EMA surveys. Average EMA survey compliance was high (**M* *= 80.21%, *SD *= 16.92%, **Mdn* *= 83.93%) with rates ranging from 38.10% to 100.00%. A Mann-Whitney U test showed that the distribution of compliance differed significantly by age group, with adolescents **(Mdn* *= 77.38%**, M* *= 73.93%, *SD* = 17.75%) having lower overall compliance than young adults **(Mdn* *= 90.48%**, M* *= 84.06%, *SD* = 15.43%; **U* *= 187.00, asymptotic **p* *= .03, *z* = −2.15, *r* = .30). A univariate linear regression that controlled for numerical age at baseline and investigated participants’ average compliance across the duration of the study, showed that neither baseline suicide attempt history (**p* *= .99) nor non-suicidal self-injury history (*p* = .42) was associated with compliance.

**Fig 3:**
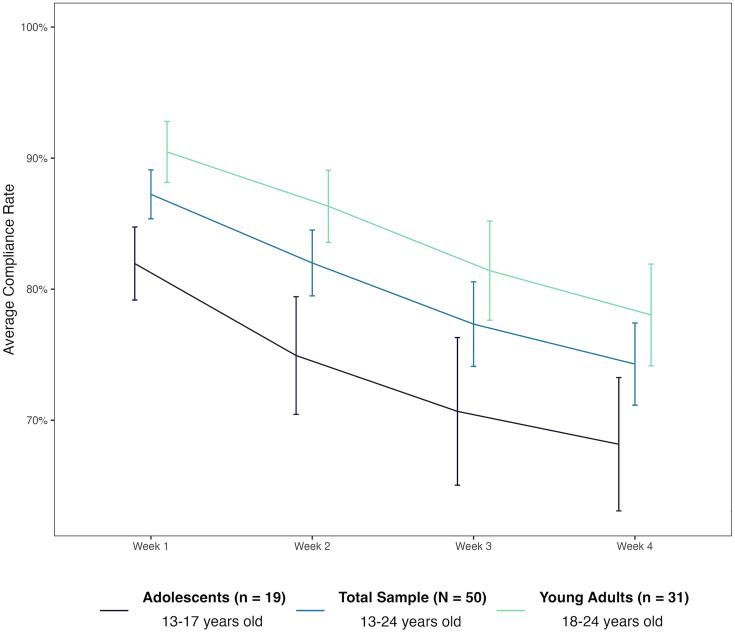
Participant compliance across the four weeks of EMA participation. Error bars represent standard errors.

Average compliance rates among all participants showed a decreasing trend across the four weeks of participation with the highest rate for Week 1 (*M* = 87.24%, *SD* = 13.21%, **Mdn* *= 92.86%), followed by Week 2 (*M* = 82.00%, *SD* = 17.77%, **Mdn* *= 88.10%), Week 3 (*M* = 77.33%, *SD* = 22.82%, *Mdn* = 85.71%), and then Week 4 (*M* = 74.29%, *SD* = 22.15%, *Mdn* = 76.19%). A one-way repeated-measures ANOVA with a Huynh-Feldt correction investigating differences in average compliance by week was significant (*F*(2.69, 131.92) = 13.45, *p* < .001, η_p_^2 ^= .22). Estimated marginal means pairwise comparisons revealed that the compliance rates of Week 2, Week 3, and Week 4 were significantly different than the compliance rate of Week 1 (*p* = .049, *p* = .003, **p* *< .001, respectively after Bonferroni correction). Similarly, the compliance rate of Week 4 was significantly different than the compliance rate of Week 2 (**p* *= .002 after Bonferroni correction). Fig 3 depicts the decrease in compliance by participants’ week of participation. A linear multilevel model with a random intercept also corroborated the negative association between weekly compliance and time (operationalized as week of participation), suggesting that participants responded to fewer EMA surveys across weeks (**b* *= −4.35, 95% CI = [−5.71, −3.00], *p* < .001).

A linear multilevel model with time (operationalized as week of participation), age group (dummy coded for adolescents), and their interaction as predictors was estimated and suggested that age group was marginally not associated with weekly compliance rate (**b* *= −9.62, 95% CI = [−19.91, 0.66], *p* = .07), while the interaction term was also not significant (**b* *= −0.34, 95% CI = −3.13, 2.46], *p* = .81). When interpreting the significance of these estimates, it is imperative to consider the small sample size at the highest level of analysis due the presence of only 19 adolescents and 31 young adults. Estimated marginal means pairwise comparisons between age group (young adults vs. adolescents) for each survey type (morning, afternoon, or evening) revealed that compliance was only significantly different for the morning surveys, with adolescents responding to fewer surveys than young adults (*p* = .002 after Bonferroni correction). Table 4 presents descriptives statistics of compliance percent overall and by survey type (morning, afternoon, evening) stratified by age group.

**Table 4:** EMA compliance percent overall and by survey type (Morning, Afternoon, Evening) split by age group.

Compliance	Total (*N *= 50)			Young adults (*n *= 31)			Adolescents (*n *= 19)		
	Range	*M* (*SD*)	*Mdn*	Range	*M* (*SD*)	*Mdn*	Range	*M* (*SD*)	*Mdn*
Overall	38.10-100.00	80.21 (16.92)	83.93	41.67-100.00	84.06 (15.43)	90.48	38.10-100.00	73.93 (17.75)	77.38
Morning	14.29-100.00	76.29 (21.76)	80.36	42.86-100.00	83.53 (17.02)	89.29	14.29-100.00	64.47 (23.85)	67.86
Afternoon	32.14-100.00	83.64 (18.79)	89.29	32.14-100.00	85.25 (17.68)	89.29	32.14-100.00	81.02 (20.69)	85.71
Evening	28.57-100.00	80.71 (18.42)	83.93	28.57-100.00	83.41 (16.48)	85.71	42.86-100.00	76.32 (20.93)	78.57

#### Safety and risk monitoring protocol.

Across the 3,369 EMA surveys completed during the study, the safety and risk protocol was activated only once. A trained RA subsequently administered the C-SSRS to the participant, whose responses categorized them as low risk. None of the evening open-ended responses where participants described their most negative event of the day (**n* *= 1,130) were flagged as high risk. The very low frequency of high risk responses might be attributable to two factors: (1) the study’s exclusion criteria, which omitted individuals with a suicide attempt in the past 90 days and thus may have limited the sample to those with less severe suicidal thoughts and behaviors; and (2) the fact that most participants were recruited through social media advertisements or LGBTQ+ community events, rather than through mental and behavioral healthcare settings, which may have limited the clinical severity of the sample. One participant was psychiatrically hospitalized during the study period. This participant was re-screened, re-consented, and re-enrolled in the study following their discharge from the hospital since they were deemed to remain eligible upon assessment.

#### Research assistant engagement with participants.

Given the intensive nature of EMA protocols, we calculated the average time research assistants (RAs) spent in direct engagement with participants as an additional indicator of study feasibility. This estimate does not include time spent on broader study logistics (e.g., IRB preparation, team meetings, or clinical supervision).

On average, RAs spent approximately 10 minutes per day per participant reviewing EMA responses, checking for safety signals, and maintaining contact via text, totaling approximately 280 minutes (4.7 hours) across the 28-day data collection period. An additional 10 minutes per day were dedicated to tracking and documentation, resulting in another 280 minutes (4.7 hours).One-time study activities included a 60-minute screener and informed consent process, a 90-minute baseline assessment, and a 30-minute exit interview. Infrequent but necessary additional tasks included approximately 30 minutes for safety check-ins and, in rare cases, 60 minutes for re-screening and re-consenting participants following psychiatric hospitalization. Taken together, the total estimated RA time per participant ranged from approximately 13–15 hours over the 28-day study period.

### Acceptability

#### Weekly acceptability feedback surveys.

Table 5 presents descriptive statistics of the weekly feedback survey questions. Overall, participants reported that the EMA surveys were highly easy to understand (**M* *= 9.28, *SD *= 0.95), captured their experiences and feelings well (**M* *= 7.42, *SD *= 1.38), and were able to be completed privately (**M* *= 8.72, *SD *= 1.58). Participants reported that the EMA surveys were moderately interesting/engaging (**M* *= 5.15, *SD *= 2.18), and not particularly boring (**M* *= 3.43, *SD *= 2.14) or annoying/disruptive (**M* *= 2.84, *SD *= 2.25). Across the 4 weeks, 44.0% of participants indicated that they had experienced technical difficulties at least once. In open-text response boxes where participants described the technical difficulty, participants mentioned various issues including challenges reading survey text when their phone was in “dark mode” and issues loading surveys. Overall, most technical difficulties referenced issues receiving survey notifications, which was usually resolved by instructing the participant to uninstall and then reinstall the MetricWire app.

**Table 5:** Descriptive statistics from the weekly acceptability feedback surveys averaged per participant.

Question^a^	Total (*N *= 50)				Young adults (*n *= 31)				Adolescents (*n *= 19)		
	Range	*M* [*SD*]/ *n* (%)	*Mdn*	Range		*M* [*SD*]/ *n* (%)	*Mdn*	Range		*M* [*SD*]/ *n* (%)	*Mdn*
Experienced Technical Difficulties^b^		22 (44.0)				14 (45.2)				8 (42.1)	
Privacy^c^	4.25- 10.00	8.72 [1.58]	9.46	6.25- 10.00		9.07 [1.18]	9.75	4.25- 10.00		8.15 [1.97]	8.50
Capture Experiences and Feelings^d^	3.75- 10.00	7.42 [1.38]	7.75	5.00- 10.00		7.56 [1.18]	7.75	3.75- 10.00		7.19 [1.67]	7.25
Easy to Understand^e^	4.25- 10.00	9.28 [0.95]	9.50	8.00- 10.00		9.51 [0.58]	9.75	4.25- 10.00		8.90 [1.30]	9.25
Annoying/Disruptive^f^	0.00- 9.50	2.84 [2.25]	2.50	0.00- 7.00		2.88 [2.00]	2.75	0.00- 9.50		2.79 [2.66]	2.50
Boring^g^	0.00- 8.75	3.43 [2.14]	3.42	0.00- 7.00		3.28 [1.94]	3.25	0.00- 8.75		3.66 [2.48]	3.75
Interesting/Engaging^h^	0.75- 10.00	5.15 [2.18]	5.50	1.67- 10.00		5.47 [2.15]	6.00	0.75- 7.00		4.62 [2.17]	5.25

^a^Questions were measured on a Likert scale (0 = Not at all – 10 = Very much), except for participants’ experiences of technical difficulties, which were measured with “Yes” or “No”. If participants indicated technical difficulties in any survey, they were coded as having had experienced technical difficulties.

^b^“I experienced technical difficulties when attempting to complete the short surveys.”

^c^“I was able to complete the short surveys in a private way, and I did not fear that others would see my responses.”

^d^“The short surveys do a good job of capturing my experiences and feelings.”

^e^“The questions were easy to understand.”

^f^“I found completing the short surveys to be annoying and/or disruptive.”

^g^“I found completing the short surveys to be boring.”

^h^“I found completing the short surveys to be interesting/engaging.”

When comparing responses to weekly feedback surveys by age group (young adults vs. adolescents), the only significant difference was on the item assessing how easy the questions were to understand. A Mann-Whitney U test showed that adolescents (**Mdn* *= 9.25, *M* = 8.90, *SD *= 1.30) reported the EMA questions to be less easy to understand than young adults (**Mdn* *= 9.75, *M = *9.51, *SD* = 0.58; **U* *= 184.50, asymptotic **p* *= .025, *z* = −2.24, *r* = .32), although both age groups still reported an overall high level of ease of understanding. Linear multilevel models with random intercepts showed that time (operationalized as week of participation) as a predictor was only positively associated with participants’ ratings of how annoying and/or disruptive the surveys were, suggesting that, over time, participants level of annoyance with the surveys significantly increased (*b* = .35, 95% CI = [0.16, 0.54], *p* < .001). No other associations between week and acceptability feedback outcomes were statistically significant.

#### Qualitative feedback from exit interviews.

Of the 50 participants, 28 (56.0%) volunteered and completed an exit interview. A QCA identified four main themes: 1) Barriers to Engagement; 2) Facilitators of Engagement; 3) Recommendations; and 4) Intervention Implications, with each theme containing several subthemes. Table 6 provides findings from the QCA including themes, subthemes, descriptions, salient quotes, and sub-theme frequencies, stratified by age group (young adults vs. adolescents).

**Table 6:** Qualitative content analysis frequencies, themes, descriptions, and quotes from exit interviews.

Themes	Sub-themes	Brief description	Total (*n *= 28)	Young Adults (*n *= 16)	Adolescents (*n *= 12)	Salient Quotes
			n (%)	*n* (%)	n (%)	
**Barriers to engagement**	Question relatability	Participants endorsed that the nature of their experiences were not well captured by study measures or protocol.	12 (42.9)	8 (50.0)	4 (33.3)	*“I don’t know, sometimes they didn’t fully describe how I was actually feeling, if that makes sense... Sometimes I felt like it wasn’t encompassing a full range.”*
	Repetitiveness	Participant described the repetitive nature of the study as having a negative impact.	24 (85.7)	14 (87.5)	10 (83.3)	*“After a week, it started feeling very repetitive, just because I was answering the same questions every day, and then it got boring after that week.*
	Schedule conflicts	Participants described being unable to answer surveys and/or were burdened by managing the survey schedule with their own personal schedules.	22 (78.6)	11 (68.8)	11 (91.7)	*When I was in school, I found that there was an issue because during my first period, my teacher takes away our phones, and so I did not have access for my phone for a large portion of the window for the first survey.”*
	EMA disruptions	Participants described unplanned or unscheduled times—not part of their normal routine—in which it is or would be disruptive to complete an EMA survey.	22 (78.6)	12 (75.0)	10 (83.3)	*“At night it can be bothersome because you just forget. Like on a Saturday, I’m out with friends and I’m like, wait, I got to go track I’m filing guys.”*
**Facilitators to engagement**	Convenient	Participants shared user-friendly and intuitive aspects of the app/interface and convenient features.	25 (89.3)	14 (87.5)	11 (91.7)	*“Overall, the app was very easy to navigate and to understand as it notified me whenever I had something to complete. I really liked how it was formatted… It was really easy to understand all the questions and to navigate throughout them. I really enjoyed it.”*
	Routine	Participants integrated the process into their daily routine, highlighting the ease and regularity of their engagement over time.	16 (57.1)	12 (75.0)	4 (33.3)	*“Near the end of the survey, it just became like a habit to do them. It was pretty easy to get into the flow of things.”*
	Engagement with research team	Participants reported positive experiences with the research team’s communication and support, highlighting balanced and responsive interactions that enhanced their engagement and connection with the study.	21 (75.0)	13 (81.3)	8 (66.7)	*Specifically also with the weekly feedback surveys and whatever, I enjoyed being able to have that chance to give some feedback and connect with you guys, and you were very involved in the process and it felt like you cared, and that was very nice.”*
	Commitment to research	Participants referred to the relevancy, saliency, and/or importance, of the research regarding mental health and/or LGBTQ+ identity.	9 (32.1)	6 (37.5)	3 (25.0)	*“I honestly enjoy the questions relating to my experiences as an LGBT person too. That’s part of the reason why I joined the study.”* *“...I really do like what you’re doing and how you’re doing this study to help people. Especially for people who don’t really talk about their mental health, doing the survey definitely will help them feel seen and it makes me feel better because I’m helping to expand it [the research].”*
	Promoting emotional awareness	Participants described their accounts of engaging in emotional awareness processes including mindfulness of present emotions and reflections on past emotional states and experiences as a result of answering survey questions, with implications for intervention.	26 (92.9)	15 (93.8)	11 (91.7)	*“It definitely allowed me to actually realize what I was feeling, and it definitely, the surveys calm me down a bit if I was like feeling really anxious or really angry or really upset and they helped me to actually like figure out, I am feeling this way and then it caused me to sometimes think about what I could do in order to stop feeling that way, or the things that were going in my life that I could change and do something about, instead of just being sad about it.”*
**Recommendations**	Personalization	Participants recommended features that helped account for barriers they experienced during the study or perceived for other participants.	15 (53.6)	10 (62.5)	5 (41.7)	*“...potentially being able to choose those times. For me, I have a weird work schedule. I work night shifts, so a lot of times, it was hard for me to be able to get up and do the morning survey because I was working until 11:00, midnight, 1:00 AM, and so, getting up for that survey at 8:00 or 9:00 AM sometimes was difficult.”*
	Question variety	Participants suggested enhancing the survey experience through a greater variety of questions.	21 (75.0)	11 (68.8)	10 (83.3)	*“I think maybe if there was more like open-ended questions. If there was more space to actually type out an answer to a question, that would maybe provide more insight…”*
	Back-button	Participants desired the ability to review and modify their answers.	6 (21.4)	3 (18.8)	3 (25.0)	*“If it’s possible, there were a few times where I was like, I clicked something then I was like, “Huh, I wonder if I can go back and change that,” and I couldn’t.”*
**Intervention implications**	Transition periods	Participants referred to periods of change or transition in which an EMA app may be useful or may have implications for future intervention.	8 (28.6)	5 (31.3)	3 (25.0)	*“When I’m in and out of the hospital I’m having to deal with my doctors, that’s hard for me. It takes a toll on you. Anything that would be difficult for other people, whether it’s grief or it’s just big changes like, I know August is coming up, so people will be starting school again, or whether it’s people are moving from high school to college, or even middle school to high school, stuff like that, big life changes. It definitely would be nice to sit and evaluate that type of stuff.”*
	Emotion tracking	Participants described that the study design could be useful to facilitate regular check-ins with their emotions.	12 (42.9)	6 (37.5)	6 (50.0)	*“...I’m very curious about watching where my mood goes and it would be cool to go back and look at all my previous answers just to see how it changes as an app in general, but I like keeping track.”*
	Risk	Participants reported being at low risk for suicidal ideation through the duration of the study.	11 (39.3)	8 (50.0)	3 (25.0)	*“They felt like they were very easy to answer because I wasn’t feeling any types of way, because I was pretty okay during the whole survey but I could see why it would make me feel not okay if I was actually not okay.”*
	Honest most of the time with exceptions	Participants expressed that they answered mostly honestly to the survey questions with some exceptions, such as depending on the contexts in which they were answering surveys that impacted their comfort in answering honestly.	4 (14.3)	1 (6.3)	3 (25.0)	*“I feel like I was... less honest with them [questions about suicidal ideation] than the other questions, just because it’s like an awkward thing to talk about. I’ve never really talked to anyone about it before. For the most part, I feel like I was able to be pretty honest.”*

During exit interviews, participants raised several factors that served as *barriers to their EMA survey completion*. Most participants (85.7%) endorsed feeling like the process – including EMA survey measures, the survey schedule, and the notifications – was repetitive. Many participants found that the EMA surveys were sometime difficult to complete due to schedule conflicts like school and work (78.6%), as well as other disruptions like vacations, family time, and electronic-free periods (78.6%). Some (42.9%) also suggested that they struggled to relate their experiences to the EMA survey items or felt that some items were incapable of capturing the full nuance of their experiences.

Participants endorsed several sub-themes related to *facilitators of their engagement*. Reflecting results from the weekly feedback surveys, during exit interviews, participants frequently (89.3%) noted that the study process was easy and convenient. Most participants (92.9%) also described that completing the EMA surveys prompted them to engage in emotional awareness that they would not have otherwise. Most of these emotional reflections were positive, with several participants describing that the act of completing the EMA surveys facilitated emotion regulation, allowing for structured reflection on emotions and experiences throughout their day. Approximately one-third of participants (32.1%) reported that their experience of completing the EMA surveys was improved by the project’s focus on SGM mental health. Participants highlighted appreciating answering questions related to their SGM identity and experiences as well as feeling positively about contributing to research that could ultimately benefit the LGBTQ+ community.

Participants also expressed three main *recommendations for improving the EMA study* process as well as the EMA app. First, most participants suggested personalizing timing of the EMA surveys (e.g., to align with participant work and school schedules; 53.6%) that could help to enhance compliance. Second, three-quarters of participants (75.0%) recommended enhancing the survey experience through a greater variety of questions to reduce repetitiveness, although several also acknowledged that they understood the repetitiveness to be a facet of EMA surveys (i.e., repeated assessment of the same construct). Third, some participants (25.4%) expressed a desire for a “back-button” to review and modify their answers especially in rare cases where they had accidentally clicked an incorrect response given that the EMA protocol did not allow participants to back-fill data as it could introduce bias regarding retrospective recall that EMA otherwise is able to avoid.

Regarding *implications for intervention*, participants provided feedback on how EMA tools like Project SPIRiT may hold promise for future EMA app-based interventions targeting minority stress and suicidal ideation in SGMY. Some participants (28.6%) emphasized that an EMA app-based intervention based on Project SPIRiT would be most helpful during transition periods or crises (i.e., transition to college, upon discharge from psychiatric hospitalization), but participants more frequently (42.9%) reported that they would welcome an EMA app-based intervention for routine emotion tracking and increased emotional awareness even during times of more stable mental health. Most participants (85.7%) felt comfortable answering all survey questions honestly, including questions assessing suicidal ideation. However, some (39.3%) noted that they were in a relatively low-risk mental health state during the study period and expressed that responding to numerous questions per day about suicidal ideation and related negative emotions might be more challenging during periods of increased mental health difficulty, such as during suicidal crises.

## Overall discussion

In this multi-phase project, we developed and then assessed the feasibility and acceptability of Project SPIRiT, an intensive smartphone-based EMA study of minority stress and suicidal ideation intensity among SGMY at high risk. In the pages below, we summarize findings from this comprehensive development and testing process and offer recommendations for future EMA research with SGMY.

In Study 1, the Project SPIRiT development process followed existing recommendations for designing and developing EMA studies [28,33], including using a multi-phase, iterative process with the ultimate goal of reducing participant burden and increasing participant compliance to the EMA protocol. We engaged community members spanning multiple domains (i.e., parents of SGMY, SGMY themselves, and experts) and integrated their feedback to develop and refine the EMA protocol and measures. Previous research highlights that incorporating community member input can improve EMA study design decisions, especially regarding the perceived relevance of potential EMA measures to participants’ lived experiences [19]. In this study, we expanded upon prior research by incorporating community member input throughout all stages of protocol development. This encompassed not only selecting, adapting, and generating EMA measures but also refining the sampling procedure, consent process, incentive structure, and safety and risk monitoring protocol. Additionally, based on community member feedback, we developed participant-facing informational materials highlighting data privacy protections, designed a study website that showcased study team members in effort to build trust with participants, and created internal study protocols to guide communication between participants and study staff during the 28-day study period. Given the sociopolitical context of the US Southeast, it was particularly important that the study was developed with insights from key community members to ensure that the EMA protocol and measures accurately reflected the experiences and needs of the target population.

In Study 2, we implemented Project SPIRiT *in situ* with 50 SGMY ages 13–24 residing in the US Southeast with histories of suicidal ideation and at least mild depressive symptoms, and assessed its feasibility and acceptability via compliance metrics, weekly feedback surveys, and a post-study exit interview. Baseline clinical severity measures showed that our inclusion criteria and recruitment strategies assembled a sample of SGMY at elevated suicide risk: almost all participants had received mental health treatment, most reported at least one mental health disorder diagnosed by a clinician, most had seriously considered suicide and engaged in non-suicidal self-injury, and more than one-third had attempted suicide. However, because we recruited participants from community settings rather than exclusively from hospitals and implemented a 90-day exclusion criterion for recent suicide attempts to ensure participant safety, our study was limited in variability and power to assess behavioral outcomes related to suicidality (e.g., suicide attempts, self-harm) during the 28-day study period. This contrasts with some studies involving higher-risk suicidal youth [13].

Compliance metrics from Project SPIRiT demonstrated the study’s high feasibility. The overall EMA compliance rate surpassed that of previous EMA studies focused on suicidality [13,18,55], particularly among young adult participants, where Project SPIRiT’s median compliance exceeded 90%. Compliance rates differed based on age group but were consistent across clinical severity characteristics. Adolescents (ages 13–17) exhibited significantly lower compliance compared to young adults (ages 18–24), a trend consistent with previous EMA research [56]. The lower compliance among adolescents compared to young adults was primarily driven by adolescents’ reduced response rates to morning surveys, which several participants attributed to phone restrictions during school hours. This finding suggests that personalized survey schedules where participants indicate daily time blocks in which they receive surveys may enhance EMA compliance among adolescents [55]. Also consistent with previous EMA research, our study observed a decline in compliance over time [18], with the highest compliance in Week 1 and the lowest in Week 4. However, even in the final week, participants completed an average of three-quarters of the EMA surveys, indicating sustained high compliance throughout the study period.

Based on responses to weekly feedback surveys and exit interviews, we attribute the high compliance in our study to several factors. First, our incentive structure was designed to motivate participants by compensating them for completing EMA surveys and offering bonuses for reaching weekly targets. Second, our internal protocols developed in Study 1 included structured communication between study staff and participants, with regular check-ins when participants missed several EMAs consecutively. We also aimed to match participants with the same research assistant throughout the study period (i.e., for baseline assessments, weekly check-in text messages, and exit interviews) to foster a sense of personal engagement with the research team. Third, several participants reported during exit interviews that they appreciated being part of an LGBTQ+ focused mental health study. Participants expressed that responding to questions about their identities and contributing to research that could benefit the LGBTQ+ community was meaningful and motivating, especially during a period of intense anti-LGBTQ+ legislative activity in the US Southeast [31]. Some participants noted that completing the EMAs felt like an outlet to have their voices heard in a climate that otherwise sought to stanch LGBTQ+ visibility. These findings underscore the importance of tailoring EMA research to the experiences and needs of the target population, in this case LGBTQ+ young people in the US Southeast, to enhance participant engagement and compliance.

Across weekly feedback surveys and exit interviews, participants consistently reported that Project SPIRiT was highly acceptable. Participants found the EMA study minimally burdensome, noting that the EMA app was easy to navigate and the survey items were quick and straightforward to complete. The convenience of the study was a key factor in its acceptability, with many participants emphasizing that it fit seamlessly into their daily routines. Additionally, the process of completing the EMAs appeared to foster greater emotional reflection, which participants largely viewed as a positive aspect of study participation. Several participants described how the structured nature of the EMA surveys helped them process their emotions and experiences throughout the day, highlighting the potential for EMA methods to serve as an intervention tool in their own right among SGMY. Overall, participants reported that Project SPIRiT was well-integrated into their daily lives without being burdensome or disruptive, further reinforcing its high level of acceptability.

### Challenges and opportunities for future EMA research with SGMY

Based on our experiences developing and testing Project SPIRiT, below we outline key considerations and practical guidance for researchers seeking to design and implement future EMA studies assessing suicidality among SGMY. We highlight strategies to navigate potential obstacles as well as opportunities to enhance the impact of this research:

#### 1. Engaging the Institutional Review Board (IRB) early in the research process given heightened concerns regarding sensitive topics and EMA research.

IRBs often express heightened concerns regarding research on sensitive topics such as suicide [57], especially when studies involve populations considered particularly vulnerable, such as SGMY [58,59]. These concerns may be amplified in EMA studies, where participants are asked to provide frequent reports on psychosocial factors (e.g., stress) and suicide-related outcomes throughout the day. IRB members, particularly those less familiar with the emerging field of smartphone-based real-time monitoring research, may be wary of such study designs and require additional clarification of measures to safeguard participant wellbeing during the study process.

To address these concerns and mitigate potential barriers to the research process, it is crucial to engage one’s IRB early in the research process and provide comprehensive information about the study’s ethical safeguards including its safety and risk management protocol. We also recommend presenting well-established evidence from previous research indicating that asking about suicide does not increase suicidal ideation [60], including in adolescent samples [61]. Additionally, we advise referring IRBs to established guidelines for the ethical inclusion of SGM populations, including SGM adolescents in research [62], including recent Department of Health and Human Services (HHS) guidelines for the ethical review and inclusion of LGBTQ+ participants in human subjects research [63]. Requesting a meeting with the IRB leadership to discuss the proposed EMA research project and to learn about any federal, state, or institutional policies that may need to be considered in the development of study materials can be an effective strategy to ensure a smooth and timely IRB process. Early engagement and transparent communication can not only facilitate a smoother review but also ensure that the study proceeds with the necessary ethical oversight and can be a way to obtain helpful feedback in tailoring study materials to be aligned with institutional IRB policies.

#### 2. Navigating parental permission requirements for research involving SGMY.

Previous research shows that requiring parental permission for research involving SGM adolescent minors can deter adolescents from participating for fear of being “outed” to their parents or fear of harm from unsupportive parents, hindering the representativeness of the study sample and thus its scientific validity [62,64]. Research ethics scholars have highlighted that the inadvertent exclusion of SGM adolescents from participating in research due to requirements of parental permission threatens the “ethical principles of beneficence and nonmaleficence, fidelity and responsibility, integrity, justice, and respect for people’s rights and dignity.” [65(p172)] However, in our study, the IRB required parental permission for participants aged 13–17, a common barrier reported among SGMY researchers conducting research on topics considered sensitive [64–66].

To address this challenge, we suggest that researchers conducting EMA research involving SGMY should continue to explore options for seeking waivers for parental consent for SGM adolescent minors where legally and ethically permissible, including following recent guidance for justifying waivers of parental permission for SGM adolescent minors to IRBs [65,67]. For investigators at institutions where waiving parental permission for adolescent minors is currently not possible, we suggest collaborating with researchers at institutions where such waivers are possible or focusing recruitment efforts on young adults (18–24 years old).

#### 3. Conducting EMA research with SGMY in a hostile sociopolitical climate.

The sociopolitical climate during the 2023–2024 legislative sessions in Tennessee and Alabama, which saw the introduction of numerous anti-LGBTQ+ laws and policies, most of which targeted youth [31,32], introduced significant challenges and ethical considerations for our study. During focus groups (Study 1), parents of SGMY expressed substantial concerns about the potential misuse of real-time data, particularly in the unlikely event of a breach of confidentiality. These concerns were heightened by the prevailing sociopolitical environment, characterized by intense public discourse on health-related data monitoring – such as the use of menstrual cycle tracking apps to surveil individuals seeking abortions [68,69]– and the surveillance of parents of SGMY, especially transgender and gender-diverse minors, as seen in Texas’s 2023 child abuse legislation targeting parents of transgender youth [70,71].

These concerns necessitated extensive efforts from our research team to develop rigorous data privacy and confidentiality protocols and to effectively communicate these measures to potential SGMY participants and their families. We created participant-facing study materials, information sheets, and a dedicated study website to ensure transparency and build trust. Additionally, the concerns expressed by parents required us to be flexible in the study development process. For instance, we decided to remove a previously proposed component of our study design – GPS monitoring through passive sensing in the smartphone app – due to significant parental concerns. Instead, we replaced this measure with two simple questions asking participants to report their location when they opened the smartphone app rather than tracking their location in real-time.

For researchers planning future EMA studies with SGMY, it is crucial to understand and account for the evolving sociopolitical climate in which the research will be conducted. Protecting participant privacy and safety during intensive longitudinal data collection is paramount and requires careful planning, flexibility, and transparent communication. Furthermore, as demonstrated in Project SPIRiT, integrating the voices of community members into the development of the EMA study protocol can ensure that the research reflects the needs and concerns of potential participants and their families, thereby enhancing the study’s ethical foundations and feasibility within the sociopolitical climate.

#### 4. Beyond assessment: Exploring the potential of Ecological Momentary Interventions (EMIs).

Future research offers a promising opportunity to extend EMA studies with SGMY by developing and testing ecological momentary interventions (EMIs) aimed at reducing suicidal thoughts and behaviors in this population [72]. Just-in-time adaptive interventions (JITAIs), a specific type of EMI, leverage real-time data to deliver tailored support when it is most needed, presenting a promising opportunity for smartphone-based suicide prevention and intervention [8]. However, despite their promise, no EMIs have been specifically developed or tested with SGMY to date – an important gap in the literature as EMA-based suicide research continues to grow. A recent review of digital health interventions for suicide prevention among SGMY from 1990 to 2023 identified only five such interventions [73], with just one involving a mobile application [74], which did not use an EMI approach.

In our study, exit interviews provided valuable insights into the potential translation of the Project SPIRiT EMA into an EMI. Participants indicated that the real-time emotion monitoring aspect of Project SPIRiT could be especially beneficial during high-risk transitional periods (e.g., starting college, after discharge from the hospital). These exit interviews also highlighted logistical considerations for future EMIs, such as the importance of schedule personalization to enhance accessibility.

To further advance suicide prevention through real-time data collection approaches, we encourage researchers to develop and test EMIs as a next step in translating assessment into intervention for SGMY at elevated suicide risk. For instance, one could envision a JITAI in which SGMY receive brief, evidence-based interventions – such as mini-exercises, videos, messages of support, crisis resources, or games derived from LGBTQ-affirmative cognitive-behavioral therapy (CBT) [75]– delivered to their smartphones at the right time, when needed. For example, such interventions could be triggered when participants report precipitating risks for increased suicidal ideation, such as heightened emotion dysregulation or immediately following a minority stress experience. As the field of real-time suicide prevention research continues to evolve, the development of such interventions represents a crucial and timely advancement in suicide prevention strategies for SGMY.

### Limitations

Several methodological limitations should be considered when interpreting this research. First, we did not psychometrically evaluate the EMA survey measures. While we integrated feedback from a comprehensive literature review and community member perspectives to select and adapt the EMA measures, it was beyond the scope of our study to establish their validity or reliability. While we tended to select items that were previously found to be valid and reliable in EMA studies assessing suicidal ideation and related psychosocial constructs [76], our momentary assessments of distal and proximal minority stressors were adapted from scales that have not been previously evaluated in EMA studies. Further, we also developed some new items for the present study based on community member feedback (i.e., items assessing exposure to negative news media) that were not psychometrically evaluated. This represents an important avenue for future study [77]. Second, the relatively small person-level sample size in Study 2 (*N* = 50) limited statistical power to discern differences across sociodemographic factors such as race, socioeconomic status, and gender when assessing feasibility and acceptability metrics. In future studies, researchers should consider recruiting larger samples to enhance statistical power as well as to assess how real-time associations may be moderated by sociodemographic factors, providing a more nuanced understanding of the momentary experiences of SGMY across axes of oppression and privilege. Third, regarding generalizability, the participants in Project SPIRiT were all recruited from the same high-stigma geographic region of the US, which may not generalize to the experiences of SGMY in other geographic contexts. Indeed, although this study focused on the US, increasing global trends in stigma and restrictive legislation targeting SGMY underscore the importance of conducting similar EMA research in other countries where the wellbeing of SGMY is likewise under threat. Future studies should aim to recruit SGMY with greater geographic variation within and outside the US to capture a wider range of social environments and experiences. This approach would also allow researchers to objectively measure structural stigma in conjunction with EMA-derived measures of minority stress, providing a more comprehensive understanding of how different contexts may influence suicide risk in the everyday lives of SGMY. Finally, also with respect to generalizability, it is important to note that the parents who participated in Study 1 were generally affirming of their SGMY children. Moreover, SGMY participants aged 13–17 in both Study 1 and Study 2 were required to obtain parental permission to participate, which likely resulted in a sample composed of youth with parents who were either explicitly affirming or at least willing to permit their child’s involvement in research addressing identity-related experiences and mental health. As such, the perspectives represented in this research may not reflect the experiences of SGMY from non-affirming families. Future research should prioritize the inclusion of families across a broader spectrum of SGM acceptance to more fully capture the diversity of family dynamics influencing SGMY mental health.

### Conclusion

Suicidal ideation among SGMY represents a significant public health and clinical concern, with nearly half of SGMY reporting recent thoughts of suicide [1,2]. While EMA methods are increasingly employed in psychological studies investigating the temporal dynamics of suicidal ideation, their application to the short-term impact of minority stress on suicidal ideation intensity among SGMY is scarce, especially in stigmatizing local contexts [25]. To address this gap, we conducted a rigorous, multi-phase, iterative development process involving multiple community members to create Project SPIRiT, a smartphone-based study deploying EMA three times daily over a 28-day period to SGMY in the US Southeast. Then, we deployed and assessed Project SPIRiT’s feasibility and acceptability in a sample of 50 SGMY in the Southeastern US with recent suicidal ideation and at least mild depression. Data from compliance metrics, weekly feedback surveys, and exit interviews showed that Project SPIRiT is a highly feasibly smartphone-based EMA protocol, with approximately 84% median compliance to the EMA surveys and very high acceptability across a variety of measures. Lessons drawn from Project SPIRiT offer valuable guidance for future researchers planning to conduct EMA research to study real-time associations between minority stress and suicidal ideation in SGMY.

## Supporting information

S1 Table Baseline assessments. (DOCX)

S2 Table EMA measures. (DOCX)
