# Supplementary material for: Development, feasibility, and acceptability of a smartphone-based ecological momentary assessment of minority stress and suicidal ideation among sexual and gender minority youth
Source: PLoS One. 2025 Aug 12;20(8):e0330204. doi: 10.1371/journal.pone.0330204 (PMC12342249; doi:10.1371/journal.pone.0330204)
Supplement: S1 Table — (DOCX) [file pone.0330204.s001.docx]

**Table S1. List of Baseline Assessments and Sources.**

| **Measure Name** | **Citation** |
| --- | --- |
| Sociodemographics | No citation. |
| Gender Minority Stress and Resilience Measure for Adolescents (GMSR-A) | Hidalgo MA, Petras H, Chen D, Chodzen G. The Gender Minority Stress and Resilience Measure: Psychometric validity of an adolescent extension. Clin. Pract. Pediatr. Psychol. 2019 Sep 1;7(3):278–90. doi:10.1037/cpp0000297. |
| Bullying and Cyberbullying | Minnesota Department of Education, Minnesota Department of Health. Being bullied for specific reasons. 2019. 13–15. (2019 Minnesota Student Survey statewide tables). Available from: <https://www.lrl.mn.gov/docs/2020/Other/200025.pdf>  Adapted from Tables 10a, 10b, and 11a. |
| Outness Inventory (OI) | Mohr J, and Fassinger R. Measuring dimensions of lesbian and gay male experience. *Meas. Eval. Couns. Dev*. 2000 Jul 1;33(2):66–90. doi:10.1080/07481756.2000.12068999. |
| Social Safety Questionnaire | Diamond LM, Alley J. Rethinking minority stress: A social safety perspective on the health effects of stigma in sexually-diverse and gender-diverse populations. Neurosci Biobehav Rev. 2022 Jul 1;138:104720. doi:10.1016/j.neubiorev.2022.104720. |
| Patient Health Questionnaire-9 (PHQ-9) | Kroenke K, Spitzer RL, Williams JB. The PHQ-9: Validity of a brief depression severity measure. J Gen Intern Med. 2001 Sep;16(9):606–13.  doi:10.1046/j.1525-1497.2001.016009606.x.  Kroenke K, Spitzer RL. The PHQ-9: A new depression diagnostic and severity measure. Psychiatr Ann. 2002 Sep;32(9):509–15.doi: 10.3928/0048-5713-20020901-06. |
| Generalized Anxiety Disorder-7 (GAD-7) | Spitzer RL, Kroenke K, Williams JBW, Löwe B. A brief measure for assessing Generalized Anxiety Disorder: The GAD-7. Arch Intern Med. 2006 May 22;166(10):1092–7. doi:10.1001/archinte.166.10.1092. |
| Substance Use | Minnesota Department of Education, Minnesota Department of Health. Use of tobacco, alcohol, marijuana and e-cigarettes. 2019. 59. (2019 Minnesota Student Survey statewide tables). Available from: <https://www.lrl.mn.gov/docs/2020/Other/200025.pdf>  Bush K, Kivlahan DR, McDonell MB, Fihn SD, Bradley KA. The AUDIT Alcohol Consumption Questions (AUDIT-C): An effective brief screening test for problem drinking. Arch Intern Med. 1998 Sep 14;158(16):1789–95. doi:10.1001/archinte.158.16.1789.  Adapted from Table 44 of the Minnesota Student Survey statewide tables. |
| Suicidal Ideation Attribution Scale (SIDAS) | The Australian National University. ANU National Centre for Epidemiology and Population Health. [cited 2022 December 1]. Suicidal Ideation Attributes Scale (SIDAS). Available from: <https://nceph.anu.edu.au/research/tools-resources/suicidal-ideation-attributes-scale-sidas>  van Spijker BAJ, Batterham PJ, Calear AL, Farrer L, Christensen H, Reynolds J, et al. The Suicidal Ideation Attributes Scale (SIDAS): Community-based validation study of a new scale for the measurement of suicidal ideation. Suicide Life Threat Behav. 2014;44(4):408–19. doi:10.1111/sltb.12084. |
| Suicidal Behavior (SITBI-R) | Fox KR, Harris JA, Wang SB, Millner AJ, Deming CA, Nock MK. Self-Injurious Thoughts and Behaviors Interview—Revised: Development, reliability, and validity. Psychol Assess. 2020;32(7):677–89. doi: 10.1037/pas0000819.  Minnesota Department of Education, Minnesota Department of Health. Self-inflicted injury; Suicidal thoughts and suicidal behaviors. 2019. 40. (2019 Minnesota Student Survey statewide tables). Available from: <https://www.lrl.mn.gov/docs/2020/Other/200025.pdf>  Modified to include questions from the Minnesota Student Survey statewide tables. |
| Non-Suicidal Self-Injury (NSSI) | Minnesota Department of Education, Minnesota Department of Health. Self-inflicted injury; Suicidal thoughts and suicidal behaviors. 2019. 40. (2019 Minnesota Student Survey statewide tables). Available from: <https://www.lrl.mn.gov/docs/2020/Other/200025.pdf>  Fox KR, Harris JA, Wang SB, Millner AJ, Deming CA, Nock MK. Self-Injurious Thoughts and Behaviors Interview—Revised: Development, reliability, and validity. Psychol Assess. 2020;32(7):677–89. doi:10.1037/pas0000819. |
| Interpersonal Needs Questionnaire-15 (INQ-15) | Van Orden KA, Cukrowicz KC, Witte TK, Joiner TE. Thwarted belongingness and perceived burdensomeness: Construct validity and psychometric properties of the Interpersonal Needs Questionnaire. Psychol Assess. 2012 Mar;24(1):197–215. doi:10.1037/a0025358. |
| Three-Item Loneliness Scale | Hughes ME, Waite LJ, Hawkley LC, Cacioppo JT. A short scale for measuring loneliness in large surveys: Results from two population-based studies. Res Aging. 2004;26(6):655–72. doi:10.1177/0164027504268574. |
| Parental Acceptance-Rejection Questionnaire (PARQ) – Child  [for 13-17 year old participants] | Rohner RP. Parental Acceptance-Rejection Questionnaire (PARQ): Test manual. In: Rohner RP, Khaleque A, editors. Handbook for the study of parental acceptance and rejection. 4th ed. Storrs, CT: Rohner Research Publications; 2005. p. 43–106. |
| Parental Acceptance-Rejection Questionnaire (PARQ) – Adult  [for 18-24 year old participants] | Rohner RP. Parental Acceptance-Rejection Questionnaire (PARQ): Test manual. In: Rohner RP, Khaleque A, editors. Handbook for the study of parental acceptance and rejection. 4th ed. Storrs, CT: Rohner Research Publications; 2005. p. 43–106. |
| Children’s Rejection Sensitivity Questionnaire (C-RS)  [for 13-15 year old participants] | Downey G, Lebolt A, Rincón C, Freitas AL. Children’s Rejection Sensitivity Questionnaire (CRSQ). Measurement Instrument Database for the Social Science; 2013. Available from: <http://www.midss.ie/> |
| Adult Rejection Sensitivity Questionnaire (RS)  [for 16-24 year old participants] | Berenson KR, Gyurak A, Ayduk Ö, Downey G, Garner MJ, Mogg K, et al. Rejection sensitivity and disruption of attention by social threat cues. Journal of Research in Personality. 2009 Dec 1;43(6):1064–72. doi:10.1016/j.jrp.2009.07.007. |
| Rosenberg Self-Esteem Scale (RSES) | Rosenberg M. Rosenberg Self-Esteem Scale. In: Society and the adolescent self-image. Princeton, NJ: Princeton University Press; 1965. |
| Multidimensional Scale of Perceived Social Support (MSPSS) | Zimet GD, Dahlem NW, Zimet SG, Farley GK. The Multidimensional Scale of Perceived Social Support. J Pers Assess. 1988;52(1):30–41. doi:10.1207/s15327752jpa5201_2. |
| Difficulties in Emotion Regulation (DERS-18) | Victor SE, Klonsky ED. Validation of a brief version of the Difficulties in Emotion Regulation Scale (DERS-18) in five samples. J Psychopathol Behav Assess. 2016 Dec 1;38(4):582–9. doi:10.1007/s10862-016-9547-9. |
