# Supplementary material for: Development, feasibility, and acceptability of a smartphone-based ecological momentary assessment of minority stress and suicidal ideation among sexual and gender minority youth
Source: PLoS One. 2025 Aug 12;20(8):e0330204. doi: 10.1371/journal.pone.0330204 (PMC12342249; doi:10.1371/journal.pone.0330204)
Supplement: S2 Table — (DOCX) [file pone.0330204.s002.docx]

**Table S2.** **EMA Measures.**

| **Construct** | **Question Stem and Item(s)** | **Response Options** | **Adapted from Existing Measure?** | **Citation(s) of Existing Measure** | **Timing of Assessment** |
| --- | --- | --- | --- | --- | --- |
| Sleep Duration and Quality | How did you sleep last night? | Continuous: 0 (very poorly) -  10 (very well) | No. | – | Morning only. |
|  | How many hours did you sleep last night? | Open text response | Yes. Based on previous study measure (see Kirshenbaum et al., 2023). | Kirshenbaum JS, Coury SM, Colich NL, Manber R, Gotlib IH. Objective and subjective sleep health in adolescence: Associations with puberty and affect. *J Sleep Res* 2023;*32*(3). [doi.org/10.1111/jsr.13805](https://doi.org/10.1111/jsr.13805) | Morning only. |
| Social Context | Who are you with right now? (check all that apply) | Categorical: No one (I am alone);  Close friend;  Group of friends;  Roommate(s);  Romantic partner;  Parent(s);  Sibling(s);  Family member(s);  Acquaintance(s);  Stranger(s);  Coworker(s), boss(es), manager(s);  Classmate(s);  Other (please describe) | No. Received feedback from focus groups. | – | Morning, Afternoon, Evening |
|  | How accepting is this person/are these people of your LGBTQ+ identity? | Continuous: 1 (very unaccepting) - 10 (very accepting) or 11 (I don’t know this person or people well enough to answer this question) | No. Received feedback from focus groups. | – | Morning, Afternoon, Evening |
| Place-Based Context | Where are you right now? | Categorical: My house or apartment (where a parent/guardian lives);  My house, apartment, or dorm (where a parent/guardian does not live);  School;  Friend’s house or dorm;  Extracurricular activity (e.g., sport, art class, club, etc.);  Religious service;  Work;  Gym;  In public (e.g., park, store, library);  Other (please specify) | No. Received feedback from focus groups. | – | Morning, Afternoon, Evening |
|  | How accepting of your LGBTQ+ identity is this place? | Continuous: 1 (very unaccepting) - 10 (very accepting) or 11 (I am not familiar enough with this place to answer this question) | No. Received feedback from focus groups. | – | Morning, Afternoon, Evening |
| Positive and Negative Affect | Indicate how much you feel right now:  annoyed  fatigued  exhausted  discouraged  resentful  uneasy  cheerful  hopeless  on edge  angry  lively  anxious  sad  worn out  vigorous (full of life) | Continuous: 0 (not at all) - 10 (extremely) | Yes. Adapted from the Profile of Mood Scale-15 (POMS-15). | Cranford JA, Shrout PE, Iida M, Rafaeli E, Yip T, Bolger N. A procedure for evaluating sensitivity to within-person change: Can mood measures in diary studies detect change reliably? *Pers Soc Psychol Bull*. 2006;32(7):917-929. doi:[10.1177/0146167206287721](https://doi.org/10.1177/0146167206287721)  Parnes JE, Mereish EH, Meisel SN, Padovano HT, Miranda R Jr. In the presence of parents: parental heterosexism and momentary negative affect and substance craving among sexual minority youth. *J Adolesc Health*. 2023;*72*(2): 230-236. | Morning, Afternoon, Evening |
| Active Suicidal Ideation Intensity | Right now, how strong is your urge to kill yourself. | Continuous: 0 (not at all) - 10 (very strong) | Yes. Based on previous EMA study measures of Passive Suicidal Ideation (see Kleiman et al., 2020; Krall et al., 2024). | Kleiman EM, Yeager AL, Grove JL, Kellerman JK, & Kim JS. Real-time mental health impact of the COVID-19 pandemic on college students: Ecological momentary assessment study. *JMIR Mentl Health*. 2020;7(12). [doi.org/10.2196/24815](https://doi.org/10.2196/24815)  Krall HR, Ruork AK, Rizvi SL, & Kleiman EM. Hopelessness as a mechanism of the relationship between physical pain and thoughts of suicide: Results from two smartphone-based real-time monitoring samples. *Cogn Ther Res*. 2024. doi.org/10.1007/s10608-024-10472-2 | Morning, Afternoon, Evening |
| Passive Suicidal Ideation Intensity | Right now, how strong is your  desire to stay alive. | Continuous: 0 (not at all) - 10 (very strong) | Yes. Based on previous EMA study measures of Passive Suicidal Ideation (see Kleiman et al., 2020; Krall et al., 2024). | Kleiman EM, Yeager AL, Grove JL, Kellerman JK, Kim JS. Real-time mental health impact of the COVID-19 pandemic on college students: Ecological momentary assessment study. *JMIR Ment Health*. 2020;7(12). [doi.org/10.2196/24815](https://doi.org/10.2196/24815)  Krall HR, Ruork AK, Rizvi SL, Kleiman EM. Hopelessness as a mechanism of the relationship between physical pain and thoughts of suicide: Results from two smartphone-based real-time monitoring samples. *Cogn Ther Res*. 2024. doi.org/10.1007/s10608-024-10472-2 | Morning, Afternoon, Evening |
| Non-Suicidal Self-Injury Ideation Intensity | Right now, how strong is your urge to hurt your body. | Continuous: 0 (not at all) - 10 (very strong) | Yes. Adapted from previous EMA study measures of Non-Suicidal Self-Injury Intensity (see Burke et al., 2021; Kiekens et al., 2023). | Burke TA, Fox K, Kautz M, Siegel DM, Kleiman E, Alloy LB. Real-time monitoring of the associations between self-critical and self-punishment cognitions and nonsuicidal self-injury. *Behav Res Ther*. 2021;*137*,103775. <https://doi.org/10.1016/j.brat.2020.103775>  Kiekens G, Claes L, Schoefs S, Kemme NDF, Luyckx K, Kleiman EM, Nock MK, Myin-Germeys. The detection of acute risk of self-injury project: protocol for an ecological momentary assessment study among individuals seeking treatment. *JMIR Res Protoc.* 2023*;12*:e46244. [doi.org/10.2196/46244](https://doi.org/10.2196/46244) | Morning, Afternoon, Evening |
| Burdensomeness | Indicate how much you feel this way right now: The people in my life would be happier without me. | Continuous: 0 (not at all) - 10 (very much) | Yes. Adapted from the Interpersonal Needs Questionnaire (INQ-15). | Van Orden KA, Cukrowicz KC, Witte TK, Joiner TE. Thwarted  belongingness and perceived burdensomeness: Construct validity and psychometric properties of the  Interpersonal Needs Questionnaire. *Psychol Assess*. 2012;24(1):197-215. doi.org/10.1037/a0025358 | Morning, Afternoon, Evening |
| Thwarted Belongingness | Indicate how much you feel this way right now: I am close to other people. | Continuous: 0 (not at all) - 10 (very much) | Yes. Adapted from the Interpersonal Needs Questionnaire (INQ-15). | Van Orden KA, Cukrowicz KC, Witte TK, Joiner TE. Thwarted  belongingness and perceived burdensomeness: Construct validity and psychometric properties of the  Interpersonal Needs Questionnaire. *Psychol Assess*. 2012;24(1):197-215. doi.org/10.1037/a0025358 | Morning, Afternoon, Evening |
| Negative Life Events | Since the last survey, have you had an issue (like a conflict, problem, or argument) with any of  the following people? | Parent/guardian;  Sibling(s);  Friend/peer;  Someone you are talking to/in a relationship with  anyone else (teacher, other adult, other family member, boss/manager, coach);  None of the above | No. Received feedback from focus groups. | – | Morning, Afternoon, Evening |
|  | How much do you think this issue with [populate response from previous question] was related to your LGBTQ+  identity? | Continuous: 0 (not at all) - 10 (very much) | No. Received feedback from focus groups. | – | Morning, Afternoon, Evening |
| Distal Minority Stress | Please check all of the things you experienced since the last survey. | I was targeted or harassed because of my LGBTQ+ identity;  I saw or heard negative, hurtful, or offensive messages or stereotypes about my LGBTQ+ identity or people with the same LGBTQ+ identity as me;  I was ignored, isolated, or made to feel invisible because of my LGBTQ+ identity; I was misunderstood because of my LGBTQ+ identity; People stared at me because of my LGBTQ+ identity; I was not accepted because of my LGBTQ+ identity; Someone made me feel uncomfortable or unsafe because of my LGBTQ+ identity;  My LGBTQ+ identity interfered with my life;  Someone made me feel less of a human because of my LGBTQ+ identity;  None of the above | Yes. Adapted from the Everyday Identity Stress Scale (EISS). | Mereish EH, Miranda R, Liu Y, Hawthorne DJ. A daily diary study of minority stress and negative and positive affect among racially diverse sexual minority adolescents. *J of Couns Psychol*. 2021;*68*(6):670–681. [doi.org/10.1037/cou0000556](https://doi.org/10.1037/cou0000556) | Morning, Afternoon, Evening |
| Proximal Minority Stress: Identity Centrality | My LGBTQ+ identity is a central part of my identity. | Continuous: 0 (not at all) - 10 (very much) | Yes. Adapted from the Lesbian, Gay, and Bisexual Identity Scale (LGBIS). | Mohr JJ, Kendra MS. Revision and extension of a multidimensional measure of sexual minority identity: The lesbian, gay, and bisexual identity scale. *J of Couns Psychol*. 2011;58:234-45. [doi.org/10.1037/a0022858](https://psycnet.apa.org/doi/10.1037/a0022858) | Morning, Afternoon, Evening |
| Proximal Minority Stress: Expectations of Rejection | Since the last survey, did you feel worried that you would be discriminated against or treated  differently because of your LGBTQ+ identity? | Continuous: 0 (not at all) - 10 (very much) | Yes. Adapted from the Negative Expectancies subscale of the Sexual Minority Adolescent Stress Inventory (SMASI). | Schrager SM, Goldbach JT, Mamey MR. Development of the sexual minority adolescent stress inventory. *Front Psychol.* 2018; 9:319.  doi.org/10.3389/fpsyg.2018.00319 | Morning, Afternoon, Evening |
| Proximal Minority Stress: Concealment | Since the last survey, did you try to hide or “play down” your LGBTQ+ identity? | Continuous: 0 (not at all) - 10 (very much) | No. Developed through input from focus groups. | – | Morning, Afternoon, Evening |
| Proximal Minority Stress: Internalized Stigma | I wish I were heterosexual and cisgender. | Continuous: 0 (not at all) - 10 (very much) | Yes. Adapted from the Internalized Homonegativity subscale of the Lesbian, Gay, and Bisexual Identity Scale (LGBIS). | Mohr JJ, Kendra MS. Revision and extension of a multidimensional measure of sexual minority identity: The lesbian, gay, and bisexual identity scale. *J Couns Psychol.* 2011;58: 234-45. [doi.org/10.1037/a0022858](https://psycnet.apa.org/doi/10.1037/a0022858) | Morning, Afternoon, Evening |
| Negative Social Media Interactions | Think about the most negative interaction with another person you had online since the last survey. Where did this occur? | Phone call;  Text message;  Social networking site (Facebook, Instagram, Snapchat, TikTok, Twitter, etc.);  FaceTime;  Zoom/Skype/Video Webcam;  Reddit;  Twitch;  Discord;  I did not have a negative interaction online since the last survey; Other [write in response] | Yes. Adapted from the Peer Interactions measures of the Girls Interactions in Real Life Study of Brain Development (see Hamilton et al., 2021). Refined with feedback from focus groups. | Hamilton JL, Do QB, Choukas-Bradley S, Ladouceur CD, Silk JS. Where it hurts the most: Peer interactions on social media and in person are differentially associated with  emotional reactivity and sustained affect among adolescent girls. *Res Child Adolesc*  *Psychopathol*. 2021;49(2):155–167. doi.org/10.1007/s10802-020-00725-5 | Morning, Afternoon, Evening |
|  | How did this make you feel? | Continuous: 0 (negative) - 10 (positive)  Note: Respondents selected number associated with an emotion valence scale of faces. | Yes. Adapted from Peer Interactions measures of the Girls Interactions in Real Life Study of Brain Development (see Hamilton et al., 2021). Refined with feedback from focus groups. | Hamilton JL, Do QB, Choukas-Bradley S, Ladouceur CD, Silk JS. Where it hurts the most: Peer interactions on social media and in person are differentially associated with  emotional reactivity and sustained affect among adolescent girls. *Res Child Adolesc*  *Psychopathol*. 2021;49(2):155–167. doi.org/10.1007/s10802-020-00725-5 | Morning, Afternoon, Evening |
| Positive Social Media Interactions | Think about the most positive  interaction with another person you had online since the last survey. Where did this occur? | Phone call;  Text message;  Social networking site (Facebook, Instagram, Snapchat, TikTok, Twitter, etc.);  FaceTime;  Zoom/Skype/Video Webcam;  Reddit;  Twitch;  Discord;  Other [write in response]; I did not have a positive interaction online since the last survey | Yes. Adapted from Peer Interactions measures of the Girls Interactions in Real Life Study of Brain Development (see Hamilton et al., 2021). Refined with feedback from focus groups. | Hamilton JL, Do QB, Choukas-Bradley S, Ladouceur CD, Silk JS. Where it hurts the most: Peer interactions on social media and in person are differentially associated with  emotional reactivity and sustained affect among adolescent girls. *Res Child Adolesc*  *Psychopathol*. 2021;49(2):155–167. doi.org/10.1007/s10802-020-00725-5- | Morning, Afternoon, Evening |
|  | How did this make you feel? | Continuous: 0 (negative) - 10 (positive)  Note: Respondents selected number associated with an emotion valence scale of faces. | Yes. Adapted from Peer Interactions measures of the Girls Interactions in Real Life Study of Brain Development (see Hamilton et al., 2021). Refined with feedback from focus groups. | Hamilton JL, Do QB, Choukas-Bradley S, Ladouceur CD, Silk JS. Where it hurts the most: Peer interactions on social media and in person are differentially associated with  emotional reactivity and sustained affect among adolescent girls. *Res Child Adolesc*  *Psychopathol*. 2021;49(2):155–167. doi.org/10.1007/s10802-020-00725-5 | Morning, Afternoon, Evening |
| Negative Media Messages | Since the last survey, did you see or read any media or news that you found upsetting or  distressing? This could include things like a social media post, a news headline, or a TV  advertisement. | Binary: 1 (yes) or 0 (no) | Yes. Adapted from previous EMA study measures of media exposure (see Kellerman et al., 2022). | Kellerman JK, Hamilton JL, Selby EA, Kleiman EM. The mental health impact of daily news exposure during the COVID-19 pandemic: Ecological momentary assessment study. *JMIR Ment Health.* 2022; 9(5):e36966. [doi.org/10.2196/36966](https://doi.org/10.2196/36966) | Morning, Afternoon, Evening |
|  | Did the upsetting news or media specifically involve your LGBTQ+ identity or members of the  LGBTQ+ community? | Binary: 1 (yes) or 0 (no) | No. | – | Morning, Afternoon, Evening |
|  | Since the last survey, how much did you think about this upsetting media or news? | Continuous: 0 (not at all) - 10 (very much) | No. | – | Morning, Afternoon, Evening |
|  | How did it make you feel? | Continuous: 0 (negative) - 10 (positive)  Note: Respondents selected number associated with an emotion valence scale of faces. | No. | – | Morning, Afternoon, Evening |
| Positive LGBTQ+ Community Connectedness | Indicate how much you agree with each statement right now. I feel good about being part of the LGBTQ community. | Continuous: 0 (strongly disagree) - 10 (strongly agree) | Yes. Adapted from the Lesbian, Gay, and Bisexual Group Identity Measure (LBGIM). | Sarno EL, Mohr JJ. Adapting the multigroup ethnic identity measure to assess LGB group identity. *Psychol Sex Orientat and Gend Divers*. 2016;3:293-303.  doi.org/10.1037/sgd0000173 | Morning, Afternoon, Evening |
| Family Relationships | Indicate how much you feel this way right now. I feel I have a strong relationship with my family. | Continuous: 0 (not at all) - 10 (very much) | Yes. Adapted from the PROMIS Pediatric Family Relationships Measure. | Bevans KB, Riley AW, Landgraf JM, Carle AC, Teneralli RE, Fiese BH,  Meltzer LJ, Ettinger AK, Becker BD, Forrest CB. Children's family experiences: development of the PROMIS® pediatric family relationships measures. *Qual Life Res.* 2017; 26(11):3011–3023.  doi.org/10.1007/s11136-017-1629-y | Morning, Afternoon, Evening |
|  | Indicate how much you feel this way right now. I feel really important to my family. | Continuous: 0 (not at all) - 10 (very much) | Yes. Adapted from the PROMIS Pediatric Family Relationships Measure. | Bevans KB, Riley AW, Landgraf JM, Carle AC, Teneralli RE, Fiese BH,  Meltzer LJ, Ettinger AK, Becker BD, Forrest CB. Children's family experiences: Development of the PROMIS® pediatric family relationships measures. *Qual Life Res.* 2017; 26(11):3011–3023.  doi.org/10.1007/s11136-017-1629-y | Morning, Afternoon, Evening |
| Peer Relationships | Indicate how much you feel this way right now. I feel accepted by other people my age. | Continuous: 0 (not at all) - 10 (very much) | Yes. Adapted from the PROMIS Pediatric Peer Relationships Scale. | DeWalt DA, Thissen D, Stucky BD, Langer MM, Morgan DeWitt, E, Irwin DE, Lai JS, Yeatts KB, Gross HE, Taylor O, Varni JW. PROMIS Pediatric Peer Relationships Scale: Development of a peer relationships item bank as part of social health measurement. *Health Psychol.* 2023;32(10):1093–1103. doi.org/10.1037/a0032670 | Morning, Afternoon, Evening |
|  | Indicate how much you feel this way right now. I am able to count on my friends. | Continuous: 0 (not at all) - 10 (very much) | Yes. Adapted from the PROMIS Pediatric Peer Relationships Scale. | DeWalt DA, Thissen D, Stucky BD, Langer MM, Morgan DeWitt, E, Irwin DE, Lai JS, Yeatts KB, Gross HE, Taylor O, Varni JW. PROMIS Pediatric Peer Relationships Scale: Development of a peer relationships item bank as part of social health measurement. *Health Psychol.* 2023;32(10):1093–1103. doi.org/10.1037/a0032670 | Morning, Afternoon, Evening |
| Romantic Relationships | Please select which applies to you: | 0 (I am not talking to someone or in a relationship),  1 (I am talking to someone or am in a relationship with 1 person),  2 (I am talking to someone or am in a relationship with more than 1 person) | No. | - | Morning, Afternoon, Evening |
|  | If yes to 1 or 2: Indicate how much you feel this way right now. I feel I have a strong relationship with.... this person (1st option)/these people (2nd option). | Continuous: 0 (not at all) - 10 (very much) | Yes. Adapted wording from the PROMIS Family/Peer Relationship Scales. | Bevans KB, Riley AW, Landgraf JM, Carle AC, Teneralli RE, Fiese BH,  Meltzer LJ, Ettinger AK, Becker BD, Forrest CB. Children's family experiences: Development of the PROMIS® pediatric family relationships measures. *Qual Life Res.* 2017; 26(11):3011–3023.  doi.org/10.1007/s11136-017-1629-y  DeWalt DA, Thissen D, Stucky BD, Langer MM, Morgan DeWitt, E, Irwin DE, Lai JS, Yeatts KB, Gross HE, Taylor O, Varni JW. PROMIS Pediatric Peer Relationships Scale: Development of a peer relationships item bank as part of social health measurement. *Health Psychol.* 2023;32(10):1093–1103. doi.org/10.1037/a0032670 | Morning, Afternoon, Evening |
|  | If yes to 1 or 2: Indicate how much you feel this way right now. I feel really important to....this person (1st option)/ these people (2nd option). | Continuous: 0 (not at all) - 10 (very much) | Yes. Adapted wording from the PROMIS Family/Peer Relationship Scales. | Bevans KB, Riley AW, Landgraf JM, Carle AC, Teneralli RE, Fiese BH,  Meltzer LJ, Ettinger AK, Becker BD, Forrest CB. Children's family experiences: Development of the PROMIS® pediatric family relationships measures. *Qual Life Res.* 2017; 26(11):3011–3023.  doi.org/10.1007/s11136-017-1629-y  DeWalt DA, Thissen D, Stucky BD, Langer MM, Morgan DeWitt, E, Irwin DE, Lai JS, Yeatts KB, Gross HE, Taylor O, Varni JW. PROMIS Pediatric Peer Relationships Scale: Development of a peer relationships item bank as part of social health measurement. *Health Psychol.* 2023;32(10):1093–1103. doi.org/10.1037/a0032670 | Morning, Afternoon, Evening |
| Hopefulness for Tomorrow | How much do you believe this statement:  Tomorrow will be a better day than today | Continuous: 0 - 100% | No. | – | Evening only. |
| Qualitative Negative Diary | In the past 24 hours, I had a negative experience regarding my LGBTQ+ identity. | Binary: 1 (yes) or 0 (no) | Yes. Adapted from the Negative Intersectional Experiences measure (see Jackson et al., 2020). | Jackson SD, Mohr JJ, Sarno EL, Kindahl AM, Jones IL. Intersectional  experiences, stigma-related stress, and psychological health among Black LGBQ individuals. *J Consult Clin Psychol.* 2020;88(5):416–428. doi.org/10.1037/ccp0000489 | Evening only. |
|  | If Yes: Briefly describe one negative event or situation that you experienced in the last 24 hours  that relates to your LGBTQ+ identity.  If No: Briefly describe one negative event or situation that you experienced in the last 24 hours  that is not related to your LGBTQ+ identity. | Write-in answer. | Yes. Adapted from the Negative Intersectional Experiences measure (see Jackson et al., 2020). | Jackson SD, Mohr JJ, Sarno EL, Kindahl AM, Jones IL. Intersectional  experiences, stigma-related stress, and psychological health among Black LGBQ individuals. *J Consult Clin Psychol.* 2020;88(5):416–428. doi.org/10.1037/ccp0000489 | Evening only. |
| Qualitative Negative Diary: Emotion Regulation | In response to this negative event, did you use any of the following strategies to regulate or change your  emotion(s)? (check all that apply) | 1 (Just experienced it);  2 (Allowed things to be exactly as they are);  3 (Chose not to deal with it);  4 (Intentionally did something to generate or increase positive emotions);  5 (Tried to fix the problem or think of a way to make things better);  6 (Kept my mind off it by doing something else);  7 (Tried to think about the problem in a different way);  8 (I was unable to stop thinking about how I felt and those thoughts kept popping up in my  mind);  9 (I've been making fun of the situation);  10 (I've been expressing my negative emotions);  11 (Tried to forget all about it);  12 (I've been denying the problem exists);  13 (Used substances);  14 (Engaged in self injury);  15 (Sought support from other people);  16 (I've been criticizing myself);  17 (None of the above);  18 I did something else to regulate or change my emotions (please describe on the next page) | No. | – | Evening only. |
| Qualitative Positive Diary | In the past 24 hours, I had a positive experience regarding my LGBTQ+ identity. | Binary: 1 (yes) or 0 (no) | Yes. Adapted from the Negative Intersectional Experiences measure (see Jackson et al., 2020). | Jackson SD, Mohr JJ, Sarno EL, Kindahl AM, Jones IL. Intersectional  experiences, stigma-related stress, and psychological health among Black LGBQ individuals. *J Consult Clin Psychol.* 2020;88(5):416–428. doi.org/10.1037/ccp0000489 | Evening only. |
|  | If yes: In 2-3 sentences or more, briefly describe one positive event or situation that you experienced in the last 24 hours  that relates to your LGBTQ+ identity.  If no: In 2-3 sentences or more, briefly describe one positive event or situation that you experienced in the last 24 hours  that is not related to your LGBTQ+ identity. | Write-in answer. | Yes. Adapted from the Negative Intersectional Experiences measure (see Jackson et al., 2020). | Jackson SD, Mohr JJ, Sarno EL, Kindahl AM, Jones IL. Intersectional  experiences, stigma-related stress, and psychological health among Black LGBQ individuals. *J Consult Clin Psychol.* 2020;88(5):416–428. doi.org/10.1037/ccp0000489 | Evening only. |
